# Nucleic Acid–Based Therapeutics in Orphan Neurological Disorders: Recent Developments

**DOI:** 10.3389/fmolb.2021.643681

**Published:** 2021-04-28

**Authors:** Olga Khorkova, Jane Hsiao, Claes Wahlestedt

**Affiliations:** ^1^ OPKO Health, Miami, FL, United States; ^2^ Center for Therapeutic Innovation and Department of Psychiatry and Behavioral Sciences, University of Miami, Miami, FL, United States

**Keywords:** orphan disorder, neurological disorder, antisense oligonucleotide, siRNA, gene therapy, noncoding RNA

## Abstract

The possibility of rational design and the resulting faster and more cost-efficient development cycles of nucleic acid–based therapeutics (NBTs), such as antisense oligonucleotides, siRNAs, and gene therapy vectors, have fueled increased activity in developing therapies for orphan diseases. Despite the difficulty of delivering NBTs beyond the blood–brain barrier, neurological diseases are significantly represented among the first targets for NBTs. As orphan disease NBTs are now entering the clinical stage, substantial efforts are required to develop the scientific background and infrastructure for NBT design and mechanistic studies, genetic testing, understanding natural history of orphan disorders, data sharing, NBT manufacturing, and regulatory support. The outcomes of these efforts will also benefit patients with “common” diseases by improving diagnostics, developing the widely applicable NBT technology platforms, and promoting deeper understanding of biological mechanisms that underlie disease pathogenesis. Furthermore, with successes in genetic research, a growing proportion of “common” disease cases can now be attributed to mutations in particular genes, essentially extending the orphan disease field. Together, the developments occurring in orphan diseases are building the foundation for the future of personalized medicine. In this review, we will focus on recent achievements in developing therapies for orphan neurological disorders.

## Introduction

In recent years, nucleic acid–based therapeutics (NBTs), including antisense oligonucleotides (ASOs), siRNA, shRNA, and viral expression constructs, are becoming more mainstream in drug development. As of November 2020, 16 NBTs have received regulatory approval from the U.S. Food and Drug Administration (FDA) and/or European Medicines Agency (EMA) (Table 1; Drugs@FDA, 2020).

**TABLE 1:** Nucleic acid–based therapeutics approved by the FDA as of November 2020.

Drug name	Type	Target	Indication	Date	Company	Delivery route
Fomivirsen (Vitravene)	RNAseH, PS ASO	UL123 gene of cytomegalovirus	Cytomegalovirus retinitis	1998	Ionis, Novartis, Abbot	Intravitreal injection
Pegaptanib (Macugen)	Aptamer, pegylated PD, 2MOE, 2 F oligo	VEGF antagonist	Age-related macular degeneration	2004	NeXstar, Gilead, OSI	Intravitreal injection
Mipomersen (Kynamro)	Rnase H, PS 2MOE ASO	ApoB100	Homozygous familial hypercholesterolemia	2013	Ionis, Kastle	SC
Talimogene laherparepvec (IMLYGIC/T-Vec/Oncovex)	Immunotherapy, herpes simplex virus 1	Tumor cell lysis, immunostimulation	Melanoma	2015	BioVex, Amgen	Injection into lesions
Eteplirsen (ExonDys51)	Exon skipping, morpholino ASO	Dystrophin (DMD)	Exon 51-related Duchenne muscular dystrophy	2016	Sarepta	IV infusion
Nusinersen (Spinraza)	Exon skipping, PS 2MOE ASO	SMN2	Spinal muscular atrophy	2016	Ionis, Biogen	IT
Voretigene neparvovec (Luxturna)	Gene therapy, AAV2 vector	RPE65	Leber congenital amaurosis	2017	Spark Therapeutics, Children's Hospital of Philadelphia	Subretinal injection
Inotersen (Tegsedi)	RNAse H, PS 2MOE ASO gapmer	TTR	Hereditary transthyretin-mediated amyloidosis	2018	Ionis, Akcea	SC
Patisiran (Onpattro)	siRNA, lipid nanoparticle	TTR	Hereditary transthyretin-mediated amyloidosis	2018	Alnylam	IV
Golodirsen (Vyondys 53)	Exon skipping, morpholino ASO	Dystrophin (DMD)	Exon 53-related Duchenne muscular dystrophy	2019	Sarepta	IV infusion
Milasen	Splice switching, PS 2MOE ASO	MFSD8	Batten disease	2019	Boston Children’s Hospital	IT
Onasemnogene abeparvovec (Zolgensma)	Gene therapy, AAV9	SMN1	Spinal muscular atrophy	2019	Novartis, AveXis	IV infusion
Givosiran (Givlaari)	siRNA, GalNac-conjugated	ALAS1	Acute hepatic porphyria	2019	Alnylam	SC
Viltolarsen (Viltepso)	Exon skipping, morpholino ASO	Dystrophin (DMD)	Exon 53-related Duchenne muscular dystrophy	2020	Nippon Shinyaku Pharma	IV
Volanesorsen (Waylivra)	Rnase H, PS 2MOE ASO	ApoCIII	Familial chylomicronaemia	2020^a^	Ionis, Akcea	SC
Lumasiran (Oxlumo)	RNAi, enhanced stabilization chemistry-GalNAc	Glycolate oxidase (HAO1)	Primary hyperoxaluria type 1	2020	Alnylam	SC

a Approved in Europe only.

Hundreds of clinical trials of NBTs with diverse disease indications and mechanisms of action are being conducted (Table 2, ClinicalTrials.gov, 2020).

**TABLE 2:** Selected nucleic acid–based therapies in clinical trials as of November 2020.

Drug name	Type	Target	Indication	Phase	Company	Delivery route
Imetelstat (GRN163 L)	Telomerase blocker, NPS-palmitoyl ASO	Telomerase activity	Cancers	3	Geron	IV
Casimersen (SRP-4045, AMONDYS 45)	Exon skipping, PMO ASO	Dystrophin (DMD)	Exon 45-related Duchenne muscular dystrophy	NDA	Sarepta	IV
SRP-5051	Exon skipping, PPMO ASO	Dystrophin (DMD)	Exon 51-related Duchenne muscular dystrophy	1/2	Sarepta	IV
Emapticap pegol (NOX-E36)	SpiegelmerAptamer	CCL2 inhibitor	Cancers, diabetes	1/2	NOXXON	SC
Olaptesed pegol (NOX-A12)	SpiegelmerAptamer	CXCL12 inhibitor	Cancers	1/2	NOXXON	IV
Cemdisiran (ALN-CC5)	siRNA, ESC-GalNAc	C5 (complement pathway)	Complement-related diseases	2	Alnylam	SC
Revusiran (ALN-TTRSC)	siRNA, PE 2OMe/2 F/GalNAc	TTR	Hereditary transthyretin-amyloidosis	2	Alnylam	SC
Fitusiran (ALN-AT3)	siRNA, ESC-GalNAc	Antithrombin	Hemophilia and rare bleeding disorders (RBDs)	3	Alnylam, Sanofi	SC
Inclisiran (Leqvio)	siRNA, ESC-GalNAc	PCSK9	Familial hypercholsterolemia	3	Alnylam, The Medicines Company, Novartis	SC
AKCEA-TTR-LRx	RNAseH, LICA GalNac-PS 2MOE ASO	TTR	Hereditary TTR amyloidosis	3	Ionis, Akcea	SC
Vupanorsen (AKCEA-angptl3-lrx)	RNAseH, LICA/GalNac- PS 2MOE ASO	ANGPTL3	Diabetes, hepatic steatosis, and hypertriglyceridaemia	2	Ionis, Akcea	SC
AKCEA-APOCIII-LRx (ISIS 678354)	RNAseH, GalNacPS 2MOE ASO	APOC-III	Hypertriglyceridemia	2	Ionis	SC
Donidalorsen (Ionis-PKK-LRx)	RNAseH, GalNacPS 2MOE ASO	Prekallikrein	Hereditary angioedema, COVID-19	1/2	Ionis	SC
Suvodirsen	RNAseH, all-PS all-FL stereopure ASO	Dystrophin	Exon 51-related Duchenne muscular dystrophy	1/2	Wave	IV
Alipogene tiparvovec (Glybera)	Gene therapy, AAV1	Lipoprotein lipase	Lipoprotein lipase deficiency	Approved by EMA	AMT, UniQure	Intramuscular injection
AT132	Gene therapy, AAV8	MTM1	X-linked myotubular myopathy	1/2	Audentes, Astellas	IV
LYS-SAF302	Gene therapy, AAVrh.10	SGSH	Sanfilippo a syndrome	1	Lysogene	Intracerebral infusion
IONIS-ENAC-2.5Rx	RNAseH, PS 2MOE ASO	Epithelial sodium channel (ENaC)	Cystic fibrosis	1	Ionis	Nebulization
STK-001	Splicing optimization, 2MOE PS ASO	SCN1A	Dravet syndrome	2	Stoke	IT
Miravirsen	LNA antagomir	miR-122 blocker	Hepatitis C	2	Santaris, Roche	IV or SC
Cobomarsen (MRG-106)	LNA antagomir	miR-155	Blood cancers	2	Miragen	IV infusion
Remlarsen (MRG-201)	LNA promiR	miRNA 29 b	Pathological fibrosis	2^a^	Miragen	Intradermal injection in biopsy site
MRG-110	LNA antagomir	miRNA-92	Heart failure and other ischemic disease	1^a^	Miragen	Intradermal injection in wound
RGLS4326	Antagomir	miR-17	Autosomal dominant polycystic kidney disease	1^b^	*Regulus*	SC
RG-012	Antagomir	miR-21	Alport syndrome	2^a^	*Regulus*, Genzyme, Sanofi	SC
MTL-CEBPA	saRNA modulator, dsRNA in SMARTICLEs	CEBPA	Hepatocellular carcinoma	2^a^	MiNA	IV
Tofersen (IONIS-SOD1Rx)	RNAseH, PS 2MOE ASO	Superoxide dismutase 1 (SOD1)	SOD1 ALS	3^a^	Ionis, Biogen	IT
IONIS-C9Rx/BIIB078	RNAseH, PS 2MOE ASO	C9ORF72	C9ORF72-ALS	1-2^a^	Ionis, Biogen	IT
ION541 (BIIB105)	RNAseH, PS 2MOE ASO	ATXN2	ALS	2	Ionis, Biogen	IT
Tominersen/Ionis-HTTRx/RG6042	RNAseH, PS-2MOE	HTT	Huntington's	3	Ionis, Roche	IT
AMT-130	AAV5-miRNA	HTT	Huntington's	1	UniQure	Convection-enhanced stereotactic neurosurgical delivery
WVE-120101 and WVE-120102	RNAseH, stereopure PS ASO	Mutant HTT	Huntington’s	2	Wave Life Sciences, Takeda	IT
ION464 (Ionis-BIIB6Rx/BIIB101)	RNAseH, PS 2MOE ASO	SNCA	Parkinson’s, multiple system atrophy	2	Ionis, Biogen	IT
Ezaladcigene resoparvovec (VY-AADC/NBIb-1817)	Gene therapy, AAV2	AADC	Parkinson’s	3	Voyager, Neurocrine	MRI-guided convective infusion
AXO-lenti-PD/OXB-102	Gene therapy, lentivirus	Tyrosine hydroxylase, cyclohydrolase 1, aromatic l-amino acid decarboxylase	Parkinson’s	1/2	Axovant	MRI-guided intracerebral infusion
AAV2-GDNF	Gene therapy, AAV2	GDNF	Parkinson’s		Brain Neurotherapy	CED-infusion into putamen with stereotactic guidance
CERE-120	Gene therapy, AAV	Neurturin	Parkinson’s	1/2	Ceregene, Sangamo	Intracerebral injection
PTC-AADC (GT-AADC, AGIL-AADC)	Gene therapy, AAV	DDC	AADC deficiency	1/2	Agilis, PTC	Intracerebral
IONIS-MAPTRx (BIIB080, ISIS 81490)	PS 2MOE ASO	MAPT	Alzheimer’s, frontotemporal degeneration	1	Ionis, Biogen	IT
AAVrh.10hAPOE2	Gene therapy, AAVrh.10hAPOE2	APOE2	Alzheimer’s with homozygous APOE4	1^a^	Weill Cornell, AD Drug Discovery Foundation	Intracisternally
ABO-202	Gene therapy, scAAV9	PPT1	Ceroid neuronal lipofuscinoses 1	1/2	Abeona, Taysha	IV + IT
AAVrh.10CUhCLN2	Gene therapy, AAVrh.10	TTP1	CLN2	1/2	Weill Cornell, NIH	Intracerebral
AT-GTX-502 (scAAV9.P546.CLN3)	Gene therapy, scAAV9	Battenin	CLN3	1/2	Amicus	IT
AT-GTX-501	Gene therapy, scAAV9	CLN6	CLN6	1/2	Amicus	IT
RGX-111	Gene therapy, AAV9	IDUA	Mucopolysaccharidosis type I	1/2	REGENXBIO	Intracisternal
SB-318	ZFN-directed gene transfer, rAAV2/6	IDUA	Mucopolysaccharidosis type I	1/2	Sangamo	IV
RGX-121	Gene therapy, AAV9	IDS	Mucopolysaccharidosis type II	1/2	REGENXBIO	Intracisternal
SB-913	ZFN-directed gene transfer, rAAV2/6	IDS	Mucopolysaccharidosis type II	1/2	REGENXBIO	IV infusion
ABO-102	Gene therapy, AAV	SGSH	Sanfilippo a syndrome	1/2	Abeona	IV infusion
rAAV2/5-hNAGLU	Gene therapy, rAAV2/5	NAGLU	Sanfilippo B syndrome	1/2	UniQure, Institut Pasteur	Intraparenchymal infusion
ABO-101 (rAAV9.CMV.hNAGLU)	Gene therapy, rAAV9	NAGLU	Sanfilippo B syndrome	1/2	Abeona	IV

a Sequential assignment or parallel assignment.

b Adaptive design.

Possible reasons for the growing interest in NBTs include their high target specificity, ability to modulate previously inaccessible drug targets, extended half-life permitting infrequent dosing, and, in the case of the central nervous system (CNS), limited systemic exposure and toxicity.

In addition to the well-studied RNAi and RNAseH-based mechanisms, NBTs can be employed to interact with other, diverse novel biological regulatory processes (Wahlestedt and Khorkova, 2017; Roberts et al., 2020). These novel mechanisms are frequently discovered in the now burgeoning field of regulatory noncoding RNA and cannot be easily modulated by traditional small-molecule therapies or monoclonal antibodies. Moreover, NBTs could be designed to highly selectively target closely related proteins, specific alleles, isoforms, and even point mutations.

Notably, due to their simplified development cycle that benefits from the rational design process, NBTs are well suited for the treatment of orphan genetic diseases. At the same time, the number of orphan diseases with known genetic origins is currently increasing very fast, thanks to improvements in genome sequencing techniques and expansion of genetic studies. An increasing proportion of “common” disease cases can now be attributed to genetic alterations in particular genes, thus increasing the NBT-amenable orphan disease roster. Notably, majority of the recently identified disease-associated DNA polymorphisms are located in the noncoding regions of the genome that are more amenable to modulation by NBTs than by small molecule therapeutics.

Counterintuitively, many of the advanced NBTs, both approved for clinical use and under development, are intended for the treatment of neurological disorders despite difficulties in delivering NBTs to the CNS that result from their inability to cross the blood–brain barrier (BBB). Currently, the solution to this problem is intracerebral, intracerebroventricular (ICV), or intrathecal (IT) administration. However, these methods are invasive, have a relatively high probability of adverse events, and require office visits and frequent monitoring, which are burdensome for patients. Consequently, extensive studies are now being carried out to develop chemical modifications, carriers, and vectors that will facilitate *trans*-BBB uptake after the less invasive systemic, subcutaneous (SC), intranasal, or oral administration. Furthermore, chemical modifications or vectorized delivery of NBTs could confer more precise tissue- and cell type–specific targeting that is beneficial in many diseases.

The current clinical stage of NBT work requires extensive innovation and improvement in genetic diagnostics, NBT manufacturing, and regulatory practices. All of those are currently in the very early stages of development and, despite some early successes reviewed below, require significant further efforts.

Taken together, the achievements in genomic and NBT technology in the orphan disease field can set a path to better treatment of “common” diseases and the future of personalized medicine. Notably, one of the NBTs approved recently, milasen, is designed to specifically target a Batten disease–causing mutation known currently in one patient (Y. Kim et al, 2019; Table 1, see section *MFSD8 Gene (CLN7)*.

As basic biology and early work leading to the current advances in the NBT field have been thoroughly reviewed elsewhere (Wahlestedt and Khorkova, 2017; Roberts et al., 2020), in this review we will focus on the most recent developments in the NBT field. We will also briefly describe novel biological mechanisms that could be modulated using NBTs and investigations into ways to improve BBB permeability and tissue- and cell type–specific targeting. As much as possible, we will use the examples of NBTs in neurological disorders. We will also briefly review the status of NBTs in some of the orphan neurological disorders. Due to space considerations, we will not describe the extensive work that is being conducted with NBTs in neurosensory disorders, recently reviewed in Moore et al. (2019). Full names and locations of the companies mentioned in the text are listed in Table 3.

**TABLE 3:** List of companies mentioned in the text.

Company name (Location)	Website
Abeona Therapeutics, Inc. (Dallas, TX)	https://www.abeonatherapeutics.com/
Agilis Biotherapeutics Inc. (now PTC, Lynnfield, MA)	
Alnylam Pharmaceuticals (Cambridge, MA)	https://www.alnylam.com/
Amicus Therapeutics (Cranberry, NJ)	https://www.amicusrx.com/
Amsterdam Molecular Therapeutics (now uniQure, Amsterdam, Netherlands)	
Apic Bio Inc. (Cambridge, MA)	https://apic-bio.com/tag/gene-therapy/
Aspa Therapeutics (Palo Alto, CA)	https://aspatx.com/
Avexis (now Novartis Gene Therapies, Bannockburn, IL)	https://www.novartis.com/our-focus/cell-and-gene-therapy
AVROBIO, Inc. (Cambridge, MA)	https://www.avrobio.com/
Axovant Gene Therapies Ltd. (now Sio Gene Therapies, New York, NY)	https://www.siogtx.com/?gclid=EAIaIQobChMIjtP-_-DS7QIVh4KGCh0EKQM1EAAYASAAEgJHxPD_BwE
Biogen (Cambridge, MA)	https://www.biogen.com/en_us/home.html
Brain Neurotherapy Bio, Inc. (Columbus, OH and Oakland, CA)	https://www.brainneubio.com/company
BridgeBio Pharma (Palo Alto, CA)	https://bridgebio.com/
Ceregene (now Sangamo, San Diego, CA)	
CuRNA /OPKO Health (Miami, FL)	http://www.opko.com/therapeutics/opko-curna/
Genzyme (now Sanofi Genzyme, Cambridge MA)	https://www.sanofigenzyme.com/
Geron Corporation (Menlo Park, CA)	https://www.geron.com/
Ionis Pharmaceuticals Inc. (Carlsbad, CA )	https://www.ionispharma.com/
Lysogene (France)	https://www.lysogene.com/
MiNA Therapeutics Ltd. (London, UK)	https://www.minatx.com/
Miragen Therapeutics Inc. (Boulder, CO)	https://www.miragen.com/
Neurocrine Biosciences (San Diego, CA)	https://www.neurocrine.com/
nLife Therapeutics, S.L. (Granada, Spain)	https://www.n-life.es/
Noxxon Pharma (Germany)	https://www.noxxon.com/
Ovid Therapeutics (New York, NY and Cambridge, MA)	https://www.ovidrx.com/science/approach/
ProQR Therapeutics NV (Leiden, Netherlands)	https://www.proqr.com/
PTC (South Plainfield, NJ)	https://www.ptcbio.com/
REGENXBIO (Rockville, MD)	https://www.regenxbio.com/
Regulus Therapeutics Inc. (Carlsbad, CA)	http://www.regulusrx.com/
Roche (Basel, Switzerland)	https://www.roche.com/
Sangamo Therapeutics, Inc. (Richmond, CA)	https://www.sangamo.com/genomic-medicines
Sarepta Therapeutics (Cambridge, MA)	https://www.sarepta.com/
Stoke Therapeutics (Bedford, MA)	https://www.stoketherapeutics.com/
Stride Bio Inc. (Durham, NC)	https://www.stridebio.com/
Taysha Gene Therapies (Dallas, TX)	https://www.tayshagtx.com/
TriLink Biotechnologies Inc. (San Diego, CA)	https://www.trilinkbiotech.com/
UniQure Biopharma (Lexington, MA)	http://www.uniqure.com/
Voyager Therapeutics (Cambridge, MA)	https://www.voyagertherapeutics.com/
Wave Life Sciences USA, Inc. (Cambridge, MA)	https://www.wavelifesciences.com/

## Innovation in Nucleic Acid–Based Therapeutics Chemistry

**FIGURE 1:**
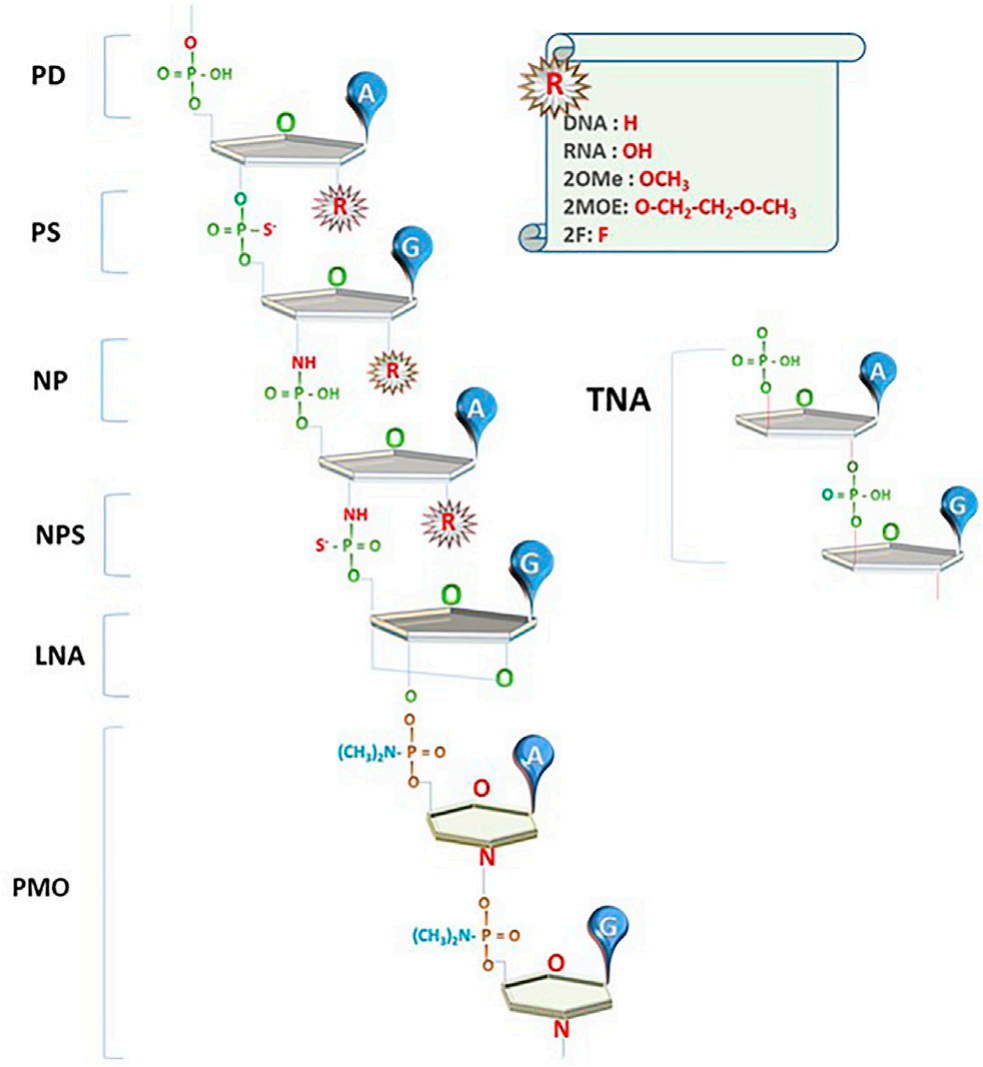
Innovations in NBT chemistry. PD: oligonucleotides with phosphodiester bond (natural DNA/RNA); PS: phosphorothioate bond; NP: N3’-->P5’ phosphoramidate bond; NPS: N3'-->P5′ thio-phosphoramidate bond; LNA: locked nucleic acid unit; PMO: phosphorodiamidate Morpholino oligomer; TNA: α-l-threose-based; A, G: nucleotide bases; R: currently used 2′O modifications of the sugar moiety.

The current successes of NBTs were made possible to a large extent due to the introduction of chemical modifications that increase nuclease resistance of DNA and RNA oligonucleotides. Phosphorothioate (PS) bonds and sugar moiety modifications, such as 2′O-methyl (2OMe), 2′-O-methoxyethyl (2MOE), fluoro (F), locked nucleic acids (LNAs), as well as phosphorodiamidate backbones and phosphorodiamidate morpholino oligomers (PMO), have already been used in approved drugs (Figure 1; reviewed in Roberts et al., 2020; Wahlestedt and Khorkova, 2017).

Although PS and otherwise chemically modified nucleic acids have extended tissue half-life in the brain due to the low nuclease content of the CSF and are readily taken up by cells, they do not penetrate BBB to a significant extent. Currently, this necessitates intracerebral, ICV, or IT administration in the clinic. However, these methods are invasive and can have severe or even life-threatening side effects. Therefore, chemical modifications that permit the administration of NBTs *via* IV, oral, inhalation, or intranasal delivery routes would be desirable and are now being actively explored.

Furthermore, chemical modifications that ensure targeted delivery of NBTs to particular tissues or cell types could reduce side effects and be beneficial in many diseases. Notably, as part of “naked” NBTs is sequestered from regulating gene expression after uptake into cells (nonproductive uptake pathway), modifications that will increase NBT escape to the productive uptake pathways will increase NBT efficiency and reduce patient exposure. In addition, dsRNA-based NBTs used to access siRNA or saRNA mechanisms require a chemical carrier for efficient delivery to cells.

Due to these considerations, the work on chemical modification of NBTs now continues to further improve NBT bioavailability, targeting to specific cell types and BBB penetration. However, every novel chemical modification has potential toxicity associated with it that may be hard to estimate using animal models. Therefore, clinical trial experience is essential in estimating the potential of novel NBT chemistries. As many other considerations, including disease prevalence, target organ, gene target selection, availability of funds, advances in manufacturing procedures, and patent positions, may influence the development of novel ASO chemistries, at this point, it is hard to predict which of them will bring the most consequential results. We briefly review some of the latest developments in NBT chemistry below.

### Phosphoramidate Backbone Chemistry

Oligonucleotides using N3'-->P5′ phosphoramidate units (NPs), where the oxygen at the 3′ position on ribose is replaced by an amine group, and N3'-->P5′ thio-phosphoramidate units (NPs), where one of the phosphate’s oxygens is replaced by sulfur, were introduced in the early 2000s (Figure 1; reviewed in Gryaznov, 2010). These compounds (termed NP-DNA or PN-chemistry) allow the formation of stable DNA and RNA duplexes with increased melting temperature and convey nuclease resistance. Although these compounds are not recognized by RNAseH, they can be used as stable RNA mimetics and in splice switching, steric blocking, and decoy applications.

Phosphoramidate chemistry is used in imetelstat, a 13-mer N3’-- > P5′ thio-phosphoramidate oligonucleotide with a covalently bound 5′ palmitoyl (C16) lipid group. Imetelstat is now in late-stage clinical trials for cancers sponsored by Geron (Table 2). Imetelstat, administered by IV infusion, is a direct competitive inhibitor of human telomerase and binds to the template region of the telomerase RNA component (Imetelstat, 2020). PN chemistry is also employed in morpholino NBTs (*Morpholino NBTs*, *NBTs in Orphan Neurological Disorders*) and stereopure NBTs (*Stereopure NBTs*).

### Morpholino Nucleic Acid–Based Therapeutics

A novel type of NBT chemistry that has already been used in clinic, called phosphorodiamidate Morpholino oligomer (PMO), is using PN bonds to link methylene morpholine rings attached to natural DNA bases (Figure 1). Two PMO drugs developed by Sarepta (eteplirsen and golodirsen) received FDA approval for the treatment of Duchenne muscular dystrophy (Table 1). Sarepta is conducting clinical trials with other PMO compounds for Duchenne: new drug application (NDA) for casimersen (Amondys 45) was submitted to the FDA in 2020; other compounds are in different stages of clinical trials. Additionally, Sarepta is developing cell-penetrating peptide-conjugated PMO (PPMO) chemistry to improve bioavailability in several disease indications. PPMO chemistry is used in SRP-5051, now in clinical trials for Duchenne (Sarepta pipeline, 2020).

Viltolarsen (NS-065/NCNP-01, Viltepso), a PMO ASO developed by Nippon Shinyaku for exon 53 skipping in Duchenne muscular dystrophy, was approved by the FDA in 2020 (Table 1; Viltolarsen, 2020).

### Nucleic Acid Aptamers

Oligonucleotide aptamers are NBTs designed to bind to protein or other molecules through mechanisms other than base pairing. One of the aptamer NBTs, pegaptanib (Macugen), a phosphodiester (PD), monomethoxy polyethylene glycol (PEG)-conjugated, oligonucleotide aptamer with 2OMe and F modifications, was approved in 2004 for the treatment of age-related macular degeneration (Table 1; Pegaptanib, 2020).

Recently, a novel aptamer NBT chemistry termed Spiegelmer has been introduced (Young et al., 2019). Spiegelmers are NBTs composed of nucleotides using L-enantiomers of the sugar moiety not occurring in natural RNA. This modification increases stability in biological matrices and decreases immunogenicity of NBTs. Noxxon is currently conducting early-stage clinical trials for two Spiegelmers: emapticap pegol and olaptesed pegol in cancers (Table 2). The Spiegelmers have demonstrated good safety profiles (Noxxon, 2020).

### α-l-Threose Oligonucleotides

Synthetic polymers of α-l-threose–based nucleic acids (TNA; Figure 1), containing a 4-carbon sugar as opposed to 5-carbon ribose found in natural nucleic acids, show strong binding affinity toward the complementary target RNAs, high nuclease resistance, and low toxicity; have easy and cost-efficient synthesis; and can be taken up by cells without transfection agents (Liu et al., 2018). TNA-based aptamers and ASOs with biological activity have been introduced. TNA ASO complementary to BcL-2 mRNA significantly reduced target mRNA and protein expression in cancer cells, as well as suppressed tumor cell growth and induced tumor cell death in carcinoma xenografts (D. Wang et al, 2019).

### Conjugated Nucleic Acid–Based Therapeutics

Conjugation of NBTs to receptor ligands or other transport molecules is employed to confer cell-type targeting and/or increased BBB permeability. Several types of conjugated NBTs have been tested in clinical trials.

#### Conjugation to Receptor Ligands

N-acetylgalactosamine (GalNac) conjugation of NBTs facilitates their uptake in the liver through binding to asialoglycoprotein receptor predominantly expressed by hepatocytes (J. Kim et al., 2019).

Alnylam used GalNac as the ligand in givosiran, an siRNA that targets mutant forms of the transthyretin gene, approved by the FDA for acute hepatic porphyria, and lumasiran, approved for primary hyperoxaluria (Table 1). Several other GalNAc-conjugated siRNA NBTs, such as cemdisiran, revusiran, fitusiran, and inclisiran, are currently in clinical trials (Table 2) (Revusiran, 2020). Revusiran is PE 2OMe/2F siRNA covalently linked at the 3′-end of its sense strand to a chain of three GalNAc residues (Table 2). The phase three trial of revusiran that targets hepatic transthyretin (TTR) for treating cardiomyopathy caused by hereditary transthyretin-mediated (hATTR) amyloidosis was discontinued after a median of 6.71 months due to observed mortality imbalance between revusiran and placebo cohorts. Although most deaths were consistent with the natural history of the disease, and no toxicity was observed in extensive animal and clinical studies, a role for revusiran could not be excluded at this time (Judge et al., 2020, *Familial Amyloid Polyneuropathy*).

Ionis’ ligand-conjugated antisense technology (LICA) involves covalent attachment of a ligand that selectively binds to receptors specific to target cells. Used in AKCEA-TTR-LRx, a GalNac-ASO also designed to inhibit production of TTR, this technology is now being tested in phase three clinical trial in patients with polyneuropathy caused by hereditary TTR amyloidosis with positive results (Table 2). Vupanorsen, a LICA/GalNac-conjugated PS 2MOE ASO that selectively inhibits ANGPTL3, for the treatment of diabetes, hepatic steatosis, and hypertriglyceridemia (Gaudet et al., 2020), and AKCEA-APOCIII-LRx, a GalNac-conjugated PS 2MOE ASO against APOC-III (Alexander et al., 2019), both delivered SC, are also showing positive results in clinical trials (Table 2).

Two patients with severe bradykinin-mediated hereditary angioedema received donidalorsen, a GalNac-conjugated PS MOE ASO against prekallikrein (PKK) developed using LICA technology platform, by SC injections for up to 8 months. Notably, this NBT is designed as a prophylactic approach in hereditary angioedema to prevent sudden painful attacks of inflammation in multiple organs that can be fatal. Donidalorsen treatment was well tolerated and accompanied by a reduction in angioedema attacks (Cohn et al., 2020). Donidalorsen is currently undergoing an investigator-initiated phase 2 clinical study in Brazil for reducing the severity of respiratory complications in patients with COVID-19 (Donidalorsen, 2020).

Conjugation to other cell type–specific ligands has been explored. Nikan et al. (Nikan et al., 2020) identified a modified neurotensin peptide that improved uptake and activity of gapmer ASOs in sortilin-expressing cells by six fold and in mouse spinal cord by 2 fold. Neurotensin conjugation also increased potency of a morpholino ASO designed to correct splicing of SMN2 in mouse cortex and striatum after ICV injection.

Covalent binding of small-molecule monoamine transporter inhibitors can also ensure cell type–specific targeting of NBTs. Indatraline, a monoamine transporter inhibitor with antidepressive properties, has been shown to bind to SERT, DAT, and NET transporters. Indatraline-conjugated ASOs (IND-ASO) designed to inhibit α-synuclein selectively reduced α-synuclein accumulation in monoamine neurons in a PD-like mouse model and elderly nonhuman primates after ICV or intranasal administration (Alarcón-Arís et al., 2020). Monoamine transporter inhibitor–conjugated NBTs are being developed by nLife Therapeutics (nLife, 2020).

#### Conjugation to Lipids

Conjugation of PS-ASOs to lipids, including palmitic acid, tocopherol, or cholesterol, was shown to enhance plasma protein binding, tissue ASO uptake, and ASO activity compared to unconjugated PS-ASOs. Lipid conjugation also facilitated PS-ASO release from endosomes after cellular uptake (F. Wang et al, 2019). These effects are of particular interest for ASO delivery to tissues such as skeletal and cardiac muscle, where high doses of unconjugated ASOs are required for therapeutic effect.

However, increased affinity of lipid-conjugated ASOs to proteins may also have a negative effect on ASO activity. Similar effects were observed *in vivo*. Palmitic acid conjugation facilitated passage of an anti-MALAT1 ASO (Palm-ASO) from plasma to tissue interstitium in mice. However, the increased accumulation of Palm-ASO in muscle interstitium, with peak at 2–4 h postinjection, did not translate into a proportional increase in ASO activity, possibly due to Palm-ASO’s tight binding to plasma proteins that rapidly carry the ASO from interstitum into lymph and back to plasma. As a result, only a small fraction of Palm-ASO entering interstitium was available for intracellular uptake occurring at a much slower rate (Chappell et al., 2020).

#### Conjugation to Other Molecules

Significant work was dedicated to ASO conjugation to cell-penetrating peptides (CPPs), also called protein transduction domains (PTDs), that are short peptides rich in basic amino acids (reviewed in Gigante et al., 2019). However, all of the studies involving CPP modifications are still in early stages of development. CPPs are frequently used in combination with other modifications.

Complex combinations of multiple delivery-improvement methods are also being tested. For example, ApoE2 encoding plasmid encapsulated in liposomes, surface-functionalized with a glucose transporter-1 targeting ligand mannose, and two cell-penetrating peptides (rabies virus glycoprotein peptide (RVG) and penetratin) was successfully delivered to mouse brain following single tail vein injection without any noticeable signs of toxicity (Arora et al., 2020).

Intranasal delivery of an AAV2 construct expressing BDNF fused with cell-penetrating peptides TAT and HA2 to a rat model of poststroke depression reversed the basal decreased sucrose consumption and prolonged immobility in the forced swimming test (C. Chen et al, 2020).

As tryptamine has been shown to effectively cross the BBB *via* active transport, Ma et al. designed tryptamine-conjugated lipidoids (NT1-lipidoids) with 14 carbons in the aliphatic tail chain mixed into BBB-impermeable lipid nanoparticles (306-O12–3) containing a PEGylated lipid DSPE-PEG2000 (Ma et al., 2020). This particle was used to deliver an anti-tau PS 2MOE ASO gapmer through tail vein injection in mice.

### Stereopure Nucleic Acid–Based Therapeutics

One of the consequences of PS modification of the oligonucleotide bonds is the introduction of a chiral center at every link of the PS chain. The binding affinity of the resulting stereoisomers to DNA/RNA, and hence their activity, is different. At the same time, standard chemical synthesis methods result in a mix of oligonucleotide stereoisomers, the exact composition of which is not easily controlled.

Wave Life Sciences has developed a scalable method of synthesizing oligonucleotides with defined stereochemistry at each PS linkage. In several completed preclinical and clinical studies, suvodirsen, the all-PS all-FL stereopure ASO designed for skipping dystrophin exon 51, was well tolerated (Table 2). Although Wave discontinued development of suvodirsen in 2019 due to lack of efficacy in an open label extension trial, other programs with stereopure NBTs are ongoing (*amyotrophic lateral sclerosis*, *frontotemporal dementia*, and *Huntington’s disease*). Wave’s current preclinical and discovery-stage programs include stereopure NBTs with partial NP backbone that were shown to have increased potency, exposure and durability in silencing, splicing, and editing applications (Suvodirsen, 2020).

In collaboration with Takeda, Wave is developing stereopure oligonucleotides with optimized profiles for CNS indications, including Alzheimer’s disease and Parkinson’s disease (Wave Programs, 2020).

### Gene Therapy

Although currently gene therapy approaches are undergoing intense development, with several approved drugs and many ongoing clinical trials (Table 1 and 2), this field has experienced a difficult start. The course of the development of therapies for Canavan disease (CD) is representative of the obstacles encountered by gene therapy and NBT fields, including lack of investment in studying pathophysiology of rare diseases and development of efficient BBB-penetrating delivery vectors (reviewed in Leone et al., 2012; *Canavan Disease*).

Also representative of the obstacles encountered by the gene therapy for orphan diseases is the development history of the first approved gene therapy NBT, alipogene tiparvovec (Glybera), an AAV1 vector carrying lipoprotein lipase, delivered by intramuscular injection. Glybera was approved in Europe in 2012 for the treatment of lipoprotein lipase deficiency (Table 2). Retrospective analysis of medical records of 19 patients treated with Glybera suggested that the treatment resulted in reduced frequency and severity of pancreatitis events and reduction in healthcare resource use for up to 6 years posttreatment (Gaudet et al., 2016). Nevertheless, in 2017, UniQure did not renew the marketing authorization for Glybera due to high cost of manufacturing and post-marketing clinical trials.

As the funding problems and diagnosis, manufacturing, and marketing difficulties still exist, the rapid progress of gene therapy may be stalled again. Recent problems in gene therapy clinical trials could also slow the development. Three patients with X-linked myotubular myopathy who received the highest dose of AT132, an AAV8 vector expressing a functional copy of the human MTM1 gene, died in the clinical trial conducted by Audentes/Astellas (Table 2). All three patients demonstrated evidence of preexisting hepatobiliary disease. The causes of deaths are being investigated (AT132, 2020).

A clinical trial of LYS-SAF302, an AAV-10 vector carrying the human N-sulfoglucosamine sulfohydrolase (SGSH) for Sanfilippo syndrome type A, was put on hold by the FDA in June 2020 following observations of localized findings on magnetic resonance imaging at the intracerebral injection sites (Table 2). In October 2020, a patient in this trial passed away. At this time, there is no evidence that the event is linked to the study drug administration (LYS-SAF302, 2020).

### Vectorized Nucleic Acid–Based Therapeutics

Despite the aforementioned difficulties, the recent achievements in gene therapy treatments led to increased interest in developing vectorized ASOs and shRNAs/miRNAs, using primarily AAV vectors (W. Chen et al, 2020).

AAV genetic material is a double-stranded DNA molecule that resides in the nucleus but is not efficiently incorporated into the host genome; hence, the danger of host mutations caused by insertion of the virus is reduced. At the same time, as the virus DNA is not replicated during cell division, it is not passed to the daughter cells, reducing the distribution of the virus. As a result, AAV constructs may require readministration, now conducted through invasive procedures. However, limited duration of the effect may provide additional safety (Hudry and Vandenberghe, 2019).

Vectorized NBTs can be administered directly to the CNS, as has been done for several gene therapy applications, but with less frequent dosing than needed for naked NBTs due to continuous NBT expression. Direct delivery to CSF or brain parenchyma can limit systemic distribution and potential host antibody development that would result in adverse effects on repeat administration. Vectorized constructs based on BBB-permeant AAV vectors can also be injected IV or administered intranasally.

Other advantages of AAV vectors include the possibility of targeting NBTs to particular cell types. Furthermore, cargo expression level by viral vectors can be modulated through the use of engineered capsids or regulatory sequences, such as RNA Pol II or Pol III promoters, enhancers, intronic sequences, and polyadenylation signals (S. Wang et al, 2019).

Further work is now being directed at optimization of the noninvasive AAV administration routes and control of localization, intensity, and timing of cargo expression.

### Gene Editing

One of the latest developments in genetic medicine is genetic editing using clustered interspaced short palindromic repeats (CRISPR)-associated protein 9 (Cas9) or transcription activator–like effector nucleases (TALENs), currently usually applied *ex vivo*. Several clinical trials employing these techniques are currently underway, mostly in cancer, HIV infection, β-thalassemia, and sickle cell disease. As these indications are beyond the scope of this review, we refer the reader elsewhere (Ernst et al., 2020).

Another genome editing method, vectorized zinc finger nucleases (ZFNs), is being tested in clinical trials in mucopolysaccharidosis and several other neurological diseases by Sangamo (*mucopolysaccharidosis*). The ZFN-gene construct is packaged into a liver-targeted AAV vector and delivered IV. Sangamo ZFNs make a double-stranded break in the DNA in a precise location in the albumin gene, where a healthy gene copy of the disease-associated gene is permanently integrated using the cell’s natural repair mechanisms (Sangamo, 2020).

Techniques utilizing the ability of endogenous human double-stranded RNA-specific adenosine deaminases (ADARs) to convert adenosine residue to inosine that is interpreted by translation machinery as G are now in preclinical stages. Qu et al. used such an approach, called “leveraging endogenous ADARs for programmable editing of RNA” (LEAPER) employing short engineered “ADAR-recruiting RNAs” (arRNAs) for recruiting native ADAR1 or ADAR2 to restore α-L-iduronidase activity in Hurler syndrome patient-derived primary fibroblast. arRNA can be delivered as a synthetic oligonucleotide or by a plasmid or viral vector. LEAPER achieves editing efficiencies of up to 80% (Qu et al., 2019).

A related approach termed “Axiomer RNA editing technology” that uses editing oligonucleotides (EONs) to guide A-to-I editing by ADARs is now being pursued by ProQR Therapeutics. The company estimates that there are over 20,000 disease-causing mutations that can be remediated by A-to-I editing (ProQR Axiomer, 2020).

Chemically optimized ASOs that can recruit endogenous human ADARs, an approach called RESTORE (recruiting endogenous ADARs to specific transcripts for oligonucleotide-mediated RNA editing), were used to repair the clinically relevant PiZZ mutation, which causes α1-antitrypsin deficiency *in vitro* (Merkle et al., 2019).

## Novel Clinical Administration Techniques

The chemical enhancements in the NBT design can be further augmented through development of novel clinical administration techniques in CNS diseases that can replace the relatively invasive direct brain administration. Novel AAV vectors that can access CNS after systemic delivery (section *Phosphoramidate Backbone Chemistry*) are one of the discoveries in this area. Extensive work is also conducted with the intranasal administration of NBTs.

It has been shown that NBTs can be aerosolized and delivered to the lung through inhalation. Delivery of a PS 2MOE ASO IONIS-ENAC-2.5Rx targeted against epithelial sodium channel (ENaC) directly to the lung *via* a Pari eFlow mesh nebulizer is being tested in a clinical trial for cystic fibrosis (Table 2). Data from the clinical trial demonstrated a more than 50% decrease in the expression of ENaC in healthy subjects (IONIS-ENAC-2.5Rx, 2020). However, brain delivery through this method would require additional modifications to increase BBB permeability.

An alternative method of enhancing intranasal delivery to the brain termed minimally invasive nasal depot (MIND) involves introducing a biodegradable NBT-containing thermo-gelling polymer gel into a pocket between nasal mucosa and skull bone in the olfactory epithelium region of the nasal cavity (Padmakumar et al., 2021). MIND delivery of AntagoNATs, PS ASOs designed to upregulate BDNF through inhibition of BDNF-AS, in a rat resulted in wide brain distribution of AntagoNAT, and induced significant and sustained upregulation of BDNF protein within the brain. The MIND technique is derived from common outpatient rhinologic procedures, uses commonly available endoscopic instrumentation, and can be easily translated into clinic. This procedure can be further enhanced by formulations that improve AntagoNAT residence time and permeation of the mucosa.

The above innovations in chemistry and delivery techniques may significantly enhance NBT use in modulating both well-known and novel disease-relevant biological mechanisms involving nucleic acids that are briefly reviewed in the next section.

## Novel Biological Mechanisms Accessible Through Nucleic Acid–Based Therapeutics

While NBTs are already widely used to achieve highly specific mRNA knockdown through RNAi/RISC and RNase H-mediated mechanisms and to modulate mRNA splicing (reviewed in Roberts et al., 2020; Wahlestedt and Khorkova, 2017), new therapeutic targets and mechanisms that can be modulated using NBTs are emerging. Some of these developments are reviewed below.

### Modulation of Splicing

As splicing mechanisms involve RNA, they can be easily modulated using NBTs. Mutations in splicing regulatory factors and aberrant mRNA splicing are known to underlie many diseases. Furthermore, progress in understanding the involvement of splicing in regulation of gene expression allows harnessing these mechanisms to correct other types of genetic alterations.

**FIGURE 2:**
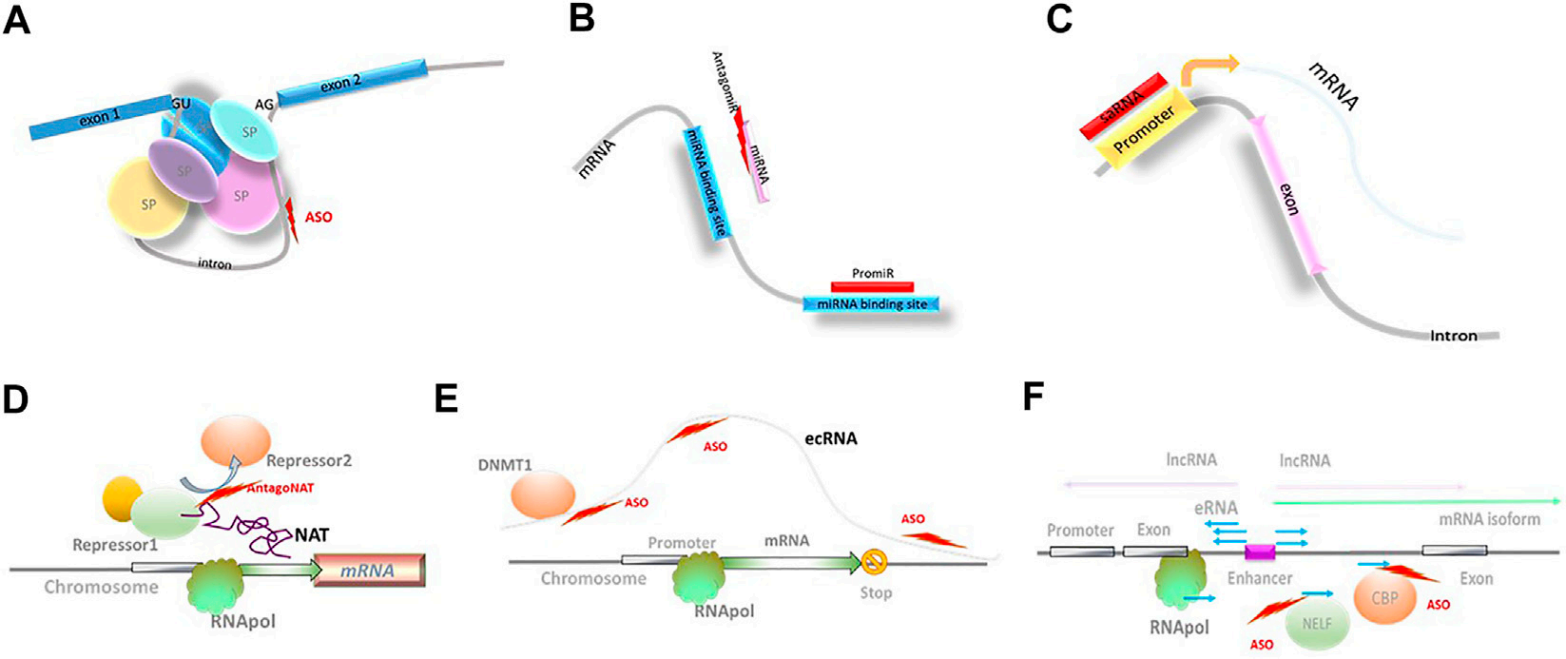
Novel biological mechanisms accessible through NBTs. **(A)** Modulation of splicing; SP: spliceosome components; ASO: antisense oligonucleotides. **(B)** Modulation of miRNA activity. **(C)** Small activating RNAs (saRNA). **(D)** Modulation of natural antisense transcript (NAT) activity; RNApol: RNA polymerase. **(E)** Extra-coding RNA (ecRNA); DNMT1: DNA methyl transferase 1. **(F)** Enhancer RNAs (eRNA); negative elongation factor complex (NELF); CBP: histone acetyltransferase CREB-binding protein.

An example of such use is nusinersen, an NBT recently approved by the FDA for the treatment of spinal muscular atrophy (SMA; Table 1, Figure 2). Three other approved NBTs (eteplirsen, golodirsen, and viltolarsen; Table 1) modulate dystrophin pre-mRNA splicing to exclude the mutated exons 51 and 53, respectively, in Duchenne muscular dystrophy. Due to particular molecular structure of dystrophin, the resulting shortened protein is still functional and can directly address the disease cause.

Another splicing-based approach utilizes the discovery of “toxic” exons naturally present in mRNAs produced by a large set of genes. Pre-mRNA molecules containing these exons cannot support translation and are destroyed through nonsense-mediated decay (Lim et al., 2020). Stoke Therapeutics is using splice-switching NBTs that can prevent the inclusion of toxic exons, and as a consequence, increase transcription of productive mRNAs. This approach, termed Targeted Augmentation of Nuclear Gene Output platform (TANGO) (Han et al., 2020), is used in STK-001, an ASO currently in clinical trial in Dravet syndrome (Table 2, *Dravet syndrome*; Stoke, 2020).

### Modulation of miRNA Activity

miRNAs that initiate mRNA silencing and modulate mRNA stability have been implicated in the development of many neurological and other diseases, and constitute an attractive target for NBT-mediated manipulation. Both synthetic miRNA mimics (promiRs) and blockers (antagomirs or miRNA sponges) have been designed, although none of the clinical stage miRNA-based programs target neurological diseases at this point (reviewed in Brites, 2020; Reza-Zaldivar et al., 2020; Innao et al., 2020; Table 2 and Figure 2; miRagen, 2020; Regulus, 2020).

### Modulation of lncRNA Activity

The recent discovery of vast noncoding RNA-mediated regulatory networks paved the way to further understanding of transcriptional and translational regulation. NBTs can be used to specifically access these novel RNA-based regulatory mechanisms briefly reviewed below.

#### Small-Activating RNA

Small-activating RNAs (saRNA) are double-stranded synthetic RNAs complementary to promoter regions (Li et al., 2006; Figure 2). The mechanism of action of saRNAs is currently not well understood and may involve epigenetic modifications at the promoter region (Wang et al., 2018). The ability of saRNA to upregulate target gene expression is utilized in MTL-CEBPA, a first-in-class saRNA therapeutic comprising amphoteric iminolipid nanoparticles called SMARTICLES and CEBPA-51, a 21-mer small activating 2′O-Me RNA oligonucleotide duplex developed by MiNA Therapeutics (Table 2). MTL-CEBPA, designed to upregulate transcription of the CEBPA gene, is in clinical trials in advanced hepatocellular carcinoma (Sarker et al., 2020).

#### Natural Antisense Transcripts

Another NBT-accessible mechanism is mediated by lncRNAs from the natural antisense transcript (NAT) class that are known to function as fine modulators of ongoing transcription. Their regulatory activity affects a single gene locus or a small subset of related genes (Figure 2). NAT-mediated regulation is present in >60% of protein-coding loci and is altered in multiple neurological disorders. Depleting NAT molecules, or blocking their interaction with epigenetic factors, DNA, or mRNA using NBTs (termed AntagoNATs), leads to upregulation of the corresponding protein-coding genes. AntagoNATs that upregulate SCN1A expression (Hsiao et al., 2016) are currently in the IND-enabling stage at CuRNA/OPKO (*Dravet Syndrome*; CuRNA/OPKO, 2020).

#### Extra-coding RNA

One of the transcription regulation mechanisms that can be targeted by NBTs are mediated by extra-coding RNAs (ecRNAs). ecRNAs are unspliced, non-polyadenylated sense transcripts that are transcribed over mRNA sequences starting upstream of their transcription start site and terminating downstream of their transcription end site, present at approximately one-quarter of all gene loci (Figure 2). It is proposed that ecRNAs promote mRNA transcription by preventing RNA Pol II from stalling (Poplawski et al., 2020). Involvement of this epigenetic mechanism could explain long-term reduction in Hdac2 expression for 16 weeks after a single ICV injection of an ASO in mice that selectively knocked down the Hdac2 ecRNA and enhanced object location memory (Poplawski et al., 2020).

Di Ruscio et al. (Di Ruscio et al., 2013) described an ecRNA transcribed from the CEBPA gene locus that binds to DNA methyltransferase DNMT1 and prevents CEBPA gene locus methylation. Deep sequencing of transcripts associated with DNMT1, combined with genome-scale methylation and expression profiling, identified numerous gene loci with potential DNMT1-binding ecRNAs. Blocking DNMT1–ecRNAs interactions using NBTs can be harnessed for gene-selective demethylation of therapeutic targets.

#### Enhancer RNA

A similar type of transcription regulation is provided by enhancer RNAs (eRNAs). Enhancers are noncoding DNA elements that include many of the typical promoter features (TATA box sequences, H3K4me1 and H3K27ac modifications, and assembly of general transcription factors (TFIID/RNAPII) and cofactors (Mediator, p300, *etc*.)), and can initiate transcription of alternatively spliced mRNA isoforms and lncRNAs (Sartorelli and Lauberth, 2020). At the same time, active enhancers are bidirectionally transcribed to produce eRNAs. Most frequently, eRNAs are defined as short (∼150 nucleotides), bidirectional, non-polyadenylated, non-spliced, capped, and unstable (Figure 2). eRNA transcription ends through early termination mechanism due to specific composition of associated elongation factors. This triggers RNA exosome recruitment and rapid degradation of eRNAs (Sartorelli and Lauberth, 2020).

eRNAs have been shown to promote gene expression through interactions with the histone acetyltransferase CBP which enhances histone acetylation at the target locus. eRNAs directly bind to BRD4 at its bromodomains, which brings BRD4 to acetylated histones in the eRNA locus and contributes to maintaining chromatin active state. eRNAs also regulate RNA Pol II pause release by being a decoy for the negative elongation factor complex (NELF) and by activating positive transcription elongation factor b complex (P-TEFb). eRNA interactions with cohesin and mediator complex may be involved in the regulation of chromatin looping required for transcriptional activation at some of the protein-coding loci (Sartorelli and Lauberth, 2020).

Due to the ease of designing NBTs that target enhancer regions/eRNAs, all above processes can now be modulated for therapeutic purposes. However, eRNA-related translational research is now in very early stages.

Although some of the chemistry improvements, enhanced delivery techniques, and novel types of biological targets reviewed above are still in the early stages of investigation, others have already yielded preclinical- and clinical-level advances in the treatment of orphan neurological disorders, which are briefly summarized below.

## NBTs in Orphan Neurological Disorders

Given that NBTs directly target DNA and RNA and have simplified development cycle compared to small-molecule therapeutics, they are particularly amenable to the treatment of orphan diseases with known genetic causes. Unique biology of each disease may require accessing different novel regulatory mechanisms and unique combinations of chemical modifications and delivery routes that are briefly described in previous sections. Several examples of application of the recent innovations in NBTs in orphan neurological diseases are given below, with the disorders that have NBTs in more advanced stages of clinical development listed first.

### Spinal Muscular Atrophy

Spinal muscular atrophy (SMA) is caused by homozygous mutations leading to reduced amounts of SMN1 protein (Messina and Sframeli, 2020). SMA prevalence is estimated at approximately 1–2 in 100,000 people, which qualifies it as an orphan disease according to the FDA definition (less than 200,000 cases in the United States or less than–1:1600). Notably, human genome contains a duplicate of the SMN1 gene (SMN2). However, normally, SMN2 produces little or no protein due to the presence of a mutation that leads to splicing out of exon 7. NBT nusinersen, recently approved by the FDA, is a PS 2MOE ASO targeting a site in intron 7 of SMN2 called ISS-N1 (Table 1). By binding to ISS-N1, nusinersen blocks splicing out of SMN2 exon 7, which increases the production of functional protein.

A gene replacement therapy onasemnogene abeparvosec (Zolgensma) was also approved by the FDA for the treatment of SMA (Table 1). Zolgensma is an AAV-9 vector carrying human SMN1 gene (Table 1).

Several other potential NBT targets in SMA are being investigated. Transcriptomic studies have identified stathmin-1 (STMN1), a tubulin-depolymerizing protein, as a potential disease modifier in several motor neuron diseases, including SMA. ICV delivery of scAAV9-STMN1 in SMA mice at postnatal day 2 increased survival and weight gain, restored microtubule networks and tubulin expression, and improved motor function, neuromuscular junction pathology, and motor neuron cell preservation (Villalón et al., 2019).

The AAV-mediated expression of myostatin propeptide (MRPO), a natural inhibitor of myostatin, administered to SMA mice *via* a single SC injection acted synergistically with PMO25, an SMN2 splice-switching morpholino ASO. By day 40 postinjection, the body weight and muscle mass were increased by 21% and 38%, respectively, and the performance in hanging wire test and treadmill exercise test improved compared to PMO25 alone (Zhou et al., 2020).

### Familial Amyloid Polyneuropathy

In patients with familial amyloid polyneuropathy (FAP) caused by transthyretin (TTR) mutations, TTR fibrils form in the CNS and peripheral organs. Thus, lowering the expression levels of both mutant and WT alleles could slow the disease progression. This approach is used in several NBTs, approved, and in late stages of development. Inotersen (Tegsedi), a 2MOE ASO designed to reduce the production of TTR, was approved by the FDA in 2018 for the treatment of all forms of TTR amyloidosis (Table 1). Patisiran (Onpattro), an anti-TTR siRNA encased in lipid nanoparticles, was approved by the FDA in 2018 (Table 1).

Development of another siRNA compound, revusiran, which was in clinical trials for hereditary ATTR amyloidosis with cardiomyopathy (hATTR-CM), was discontinued in October 2016 (Table 2; section 1.7.1, Judge et al., 2020).

### Amyotrophic Lateral Sclerosis and Frontotemporal Dementia

Amyotrophic lateral sclerosis (ALS) is a rare disease characterized by degeneration of neurons in the spinal cord and brain, and severe muscle atrophy. In majority of ALS patients, the first neurological symptoms appear in their 50s and 60s. Ninety percent of people with ALS die within five years of symptom onset. ALS affects approximately 20,000 people in the U.S. frontotemporal dementia (FTD), and ALS overlaps clinically, genetically, and pathologically. Genetic causes of these disorders are currently known only in approximately 10 percent of cases that are attributed to approximately 30 genes (Mejzini et al., 2019). Several of the genetic causes and risk factors of ALS/FTD that are now being addressed using NBTs are reviewed below.

#### SOD1-ALS

An estimated 20 percent of known-cause ALS cases result from toxic gain-of-function mutations in superoxide dismutase 1 (SOD1) gene. Disease-causing mutations are thought to increase the propensity of SOD1 to aggregate or perturb its intracellular localization signals, which leads to the formation of misfolded SOD1 aggregates in the nuclei of glial cells in the spinal cord (Pansarasa et al., 2018). As SOD1 mutations generate toxic protein, reduction in SOD1 levels is considered a desirable therapeutic target.

Tofersen, a PS 2MOE ASO delivered IT, is designed to degrade SOD1 RNA through RNAseH mechanism (Table 2). Phase I/II clinical trial in SOD1-ALS patients showed a reduction in SOD1 protein and a trend toward slower clinical decline. Lumbar puncture–related adverse events were observed in most participants (Miller et al., 2020). Tofersen is currently in a phase three clinical trial in SOD1-ALS patients with data expected in 2021.

Multiple other NBT approaches for SOD1-ALS are now in earlier stages of development. Voyager is conducting preclinical investigations of an AAV vector carrying an miRNA expression cassette (VY-SOD101) to silence SOD1 delivered *via* a single IT injection (Voyager, 2020). AVXS-301, a short hairpin RNA against SOD1, is now also in preclinical studies (Avexis, 2020). Apic Bio is in the IND-enabling stage of preclinical studies with APB-102, an anti-SOD1 miRNA carried by an AAV vector, to be delivered through single IT injection for the treatment of SOD1 ALS (Apic Bio, 2020).

An approach that does not involve direct brain administration was tested by Keeler et al. They injected an AAV vector expressing artificial microRNA targeting the SOD1 gene (AAV-miRSOD1) in the tongue and intrapleural space of SOD1G93A mice. The treatment was followed by systemic silencing of SOD1 which prolonged survival by approximately 50 days. Histologically, there was preservation of the neuromuscular junctions in the diaphragm as well as of the number of axons in the phrenic and hypoglossal nerves (Keeler et al., 2019).

Bravo-Hernandez et al. (Bravo-Hernandez et al., 2020) reported the use of a newly designed subpial injection device to deliver a single injection at cervical and lumbar levels of an AAV9 vector expressing an anti-SOD1 shRNA. The treatment was administered immediately before symptom onset in SOD1-ALS mice and induced long-term suppression of motoneuron disease. The device was also effective in larger animal models (adult pigs and nonhuman primates) where it produced homogeneous cargo delivery throughout the cervical spinal cord.

In a different approach, administration of an ASO inhibitor of miR-129-5p to an ALS animal model, SOD1 (G93A) mice, resulted in a significant increase in survival and improved the neuromuscular phenotype in treated mice (Loffreda et al., 2020).

#### C9ORF72-ALS

Another common cause of ALS is toxic gain-of-function mutations in C9ORF72 gene that encodes a putative guanine exchange factor (C9ORF72-ALS). Pathogenic mutations in C9ORF72 result in hexanucleotide repeat expansion that leads to formation of intranuclear RNA foci and noncanonical repeat-associated non-AUG translation that produces aggregation-prone dipeptide repeat proteins. Mutations in C9orf72 account for more than 30% of ALS cases with known genetic causes.

NBTs targeting C9ORF72-ALS include IONIS-C9Rx, a PS 2MOE ASO, designed to target mutant C9ORF72 RNA that is currently in clinical trials for C9ORF72-ALS conducted by Ionis/Biogen (Table 2) (IONIS, 2020). Wave Life Sciences/Takeda is investigating the use of WVE-004, a stereopure ASO, to selectively silence the repeat-containing transcripts in C9orf72 in ALS and FTD. The studies are currently in the preclinical stage (Wave Life Sciences, 2020). Sangamo/Pfizer is conducting preclinical studies with engineered zinc finger proteins (ZFPs) that also selectively target C9ORF72 allele with repeat expansion (Sangamo C9ORF72, 2020).

Extensive preclinical work is conducted to investigate the possibility of using vectorized NBT constructs in C9ORF72-ALS to reduce patient exposure to IT injections. AAV5 constructs delivering anti-C9orf72 miRNAs reduced the accumulation of repeat-containing C9orf72 transcripts and RNA foci in neurons of ALS mice (Martier et al., 2019). Additional earlier work exploring shRNA or miRNA vectorized constructs to knockdown SOD1 and C9ORF72 mRNA, as well as *ex vivo* gene therapy approaches, is reviewed in Cappella et al. (2019).

#### Other ALS-Linked Genes

Several other targets in cellular pathways involved in ALS pathogenesis are being investigated. Mutations in TARDBP (TDP-43), FUS, OPTN, VCP, UBQLN2, ATXN2, and TBK1 genes have been shown to cause ALS. Other genes associated with ALS/FTD development include multiple heterogeneous nuclear ribonucleoproteins (hnRNPs), such as hnRNP E2, hnRNP A1, R, Q, D, L, and I. All these proteins, predominantly associated with mRNA transcription and processing, are found in intracellular inclusions that are observed in majority of ALS and FTLD cases. For example, TDP-43, involved in repressing non-conserved cryptic events and nonhomologous end joining pathways of double-strand break repair, is a major component of the neuronal inclusion in ALS.

Ataxin 2 (ATXN2) has been shown to modulate TDP-43 toxicity. Mutations in ATXN2 cause one of the genetic forms of ALS (Sproviero et al., 2017). As aggregates of TDP-43 protein in motor neurons are seen in the majority of ALS cases, ATXN2 reduction would be beneficial in the sporadic ALS as well. ION541, a PS 2MOE ASO targeting ATXN2, administered IT, is currently in phase 2 clinical studies (Table 2).

Aberrant splicing of the microtubule-associated protein tau (MAPT) that leads to imbalance of the 3- and 4-repeat isoforms of this protein, accounts for up to 10% of all FTD cases. The imbalance leads to the formation of insoluble, hyperphosphorylated clusters within filaments (Bampton et al., 2020).

ALS-causing mutations in FUS cause FUS aggregation in motor neurons and lead to loss of splicing function. hnRNP A1 and FUS also enhance telomerase and topoisomerase 1 activity that prevents potentially harmful R-loop formation during transcription (Bampton et al., 2020). Overall, reduction of FUS protein in a FUS-ALS mouse model prevented motor neuron loss and may reverse or prevent disease progression in FUS–ALS patients. ION363 is a PS 2MOE ASO targeting FUS expression which is being developed for the treatment of FUS-associated ALS by Ionis (IONIS, 2020).

It has been shown that accumulation of mutant SOD1 aggregates activates the cellular unfolded protein response/integrated stress response system which suppresses global protein synthesis. This suppression can be inhibited by GADD34. This in turn could lead to response system overload and ultimately to apoptosis. In neonatal G93A and G85R mtSOD1 mice, an intravenous injection of GADD34 shRNA expressed by AAV9 vector led to increased survival and reduction in SOD1 aggregates, astrocytosis, and microgliosis (Ghadge et al., 2020).

Matrix metalloproteinase-9 (MMP9), expressed predominantly by fast motor neurons and thought to precondition them to become highly vulnerable to ALS triggers, is a potential target for the treatment of sporadic ALS. In rNLS8 mice, a sporadic ALS model where neurodegeneration is triggered by TDP-43 mislocalization and aggregation, intramuscular injection of AAV9-shRNA, ICV injection of PS MOE ASO, or genetic knockout of MMP9 preserved motor neuron counts and muscle function. Similar effects were observed in SOD1G93A mouse. Notably, as opposed to AAV9-shRNA–treated rNLS8 mice, the ASO-treated animals and Mmp9^−/−^ mice exhibited seizures and reduced survival rate, possibly due to MMP9 reduction outside of motor neurons (Spiller et al., 2019).

### Huntington’s Disease

Huntington’s disease (HD) is caused by a CAG repeat expansion in exon 1 of the huntingtin (HTT) gene that leads to the insertion of a polyglutamine (PolyQ) tract in HTT protein. The number of CAG repeats in germline and somatic cells increases with age. HD prevalence is estimated at approximately 40,000 individuals in the United States (Marxreiter et al., 2020). As HD is caused by a toxic version of HTT protein, knockdown of HTT or skipping expanded repeats during splicing constitutes desirable therapeutic targets. Tominersen is a PS-PD 2MOE ASO designed to knockdown both healthy and mutated copies of HTT mRNA (Table 2). A phase three trial of tominersen administered IT in HD that had enrolled 791 patients acrossapproximately 100 sites around the world was halted in March 2021 on the recommendation of the study monitoring board. The board did not identify any new or emerging safety signals (Tominersen, 2021).

A similar approach to knockdown HTT, but using an AAV vector with a miRNA expression cassette (VY-HTT01) delivered surgically through MRI-guided convective infusion, is being developed by Voyager/Sanofi Genzyme. IND for the first in human trial was submitted in September 2020 (VY-HTT01, 2020). Another company developing a nonselective knockdown of HTT is uniQure Biopharma (Table 2). UniQure has initiated a phase 1/2 trial of AMT-130, an AAV5 vector carrying an artificial micro-RNA, delivered through a single administration of through MRI-guided, convection-enhanced stereotactic neurosurgical delivery directly into the striatum (AMT-130, 2020).

An alternative approach to HD treatment is allele-specific HTT knockdown, which is potentially safer than the global reduction of HTT levels. Wave Life Sciences/Takeda is conducting clinical trials of a full PS stereopure ASOs WVE-120101 and WVE-120102 delivered IT (Table 2). The compounds are selectively targeting mutant HTT SNPs rs362307 and rs362331, respectively, without affecting the expression of the healthy allele. Another allele-selective candidate, WVE-003, which is designed to selectively target an undisclosed SNP (SNP3), is now in the preclinical stage. The 3 compounds can potentially provide allele-selective treatments for up to 80% of people with HD (Wave Life Sciences, 2020). In an alternative strategy for allele-specific knockdown, Sangamo/Evotec is investigating engineered zinc finger proteins (ZFPs) to target the pathogenic CAG repeat selectively without affecting the healthy allele. When delivered by a viral vector in three HD mouse models, ZFP-TFs demonstrated repression of pathogenic allele, no significant effect on healthy allele, and improvements in disease phenotype for at least nine months in the mouse brain (Zeitler et al., 2019, section *Morpholino NBTs*).

A different therapeutic strategy, now also in the preclinical stage, is used by ProQR. Toxic N-terminal fragments generated by mutant HTT cleavage cascade triggered by caspase-6–generated cut at position D586 are considered major mediators of the pathogenesis. Mutant HTT cleavage in turn drives caspase-6 activity in a feed-forward loop. QRX-704 is an ASO designed to activate an alternative HTT splice variant (HTT Δ12) that does not contain the HTT586 cleavage site. In YAC128 mouse model of HD, QRX-704 administered ICV-activated formation of HTTΔ12 with no major change to global protein folding and biochemistry after HTT exon 12 truncation (QRX-704, 2020).

While still in early preclinical stages, neuroepigenetic targets that can be easily accessed through NBTs that allow more targeted interventions than HDAC inhibitors or DNA-binding drugs are also being actively explored in HD. It has been shown that the presence of HTT mutation affects expression of multiple miRNAs and lncRNAs (Lee et al., 2013; Chen et al., 2018). Recent mouse data obtained at presymptomatic disease stages suggest that downregulation of the markers of mature medium spiny neurons, selectively affected in HD, including dopamine D1 receptor, dopamine D2 receptor, RGS9, and DARPP32, significantly precedes neuronal death (Francelle et al., 2017). Furthermore, active chromatin marker H3K27ac was depleted at neuronal super-enhancers in HD compared to controls. This was associated with depletion of RNAPII and reduced eRNA synthesis at these super-enhancers, as well as generalized downregulation of neuronal identity genes developmental arrest at an early step of neuronal maturation.

In contrast, GWAS studies indicate that H3K27ac and transcription were increased at glial-specific enhancers. Overall, genes upregulated in HD brain are enriched in immune and developmental genes (Francelle et al., 2017).

### Parkinson’s Disease

Parkinson’s disease (PD) is characterized by loss of neurons and decrease in dopamine levels in the brain, leading to tremors and movement problems (Poewe et al., 2017). Although PD that affects approximately 1 million people in the United States and 7–10 million people worldwide does not qualify as orphan disease under FDA definition, the genetic causes of disease are known in less than 10% of PD cases. Furthermore, the genetic cases are traced to multiple genes, such as alpha-synuclein (SNCA), leucine-rich repeat kinase 2 (LRRK2), PARK7, PINK1, or PRKN, which brings the prevalence of genetic subtypes into orphan disease range. In 1–2% of people with PD, the disease is caused by homozygous mutations in SNCA or LRRK2. Several risk modifier genes such as glucocerebrosidase (GBA) have also been identified. Although dopamine replacement and deep brain stimulation are being successfully used to control PD symptoms, they do not address the underlying disease causes or stop the progression of the disease. Due to unmet medical need, many of the known PD-associated genes are now being targeted by NBTs or gene therapy treatments (Nakamori et al., 2019).

#### Alpha-Synuclein

The normal physiological function of SNCA likely involves regulation of the amount of SNARE complex, which controls the release of neurotransmitters. Mutations in SNCA that affect protein folding can cause its self-assembly into fibrillar aggregates and formation of insoluble inclusions. Accumulation of SNCA aggregates in neurons and glial cells is one of the pathological hallmarks of PD (J. Du et al, 2020). Reduction of SNCA expression has been shown to reduce the formation of aggregates and ameliorate the disease in animal models. ION464, a PS 2MOE ASO targeting SNCA, is currently in clinical trials for PD and multiple system atrophy conducted by Ionis/Biogen (Table 2). Sangamo is currently in the IND-enabling stage of the preclinical studies of ZFP-TFs for repressing the expression of SNCA (Sangamo, 2020).

Indatraline-conjugated ASO (IND-ASO), designed to inhibit SNCA, improved dopamine neurotransmission in a PD-like mouse model and elderly nonhuman primates after ICV and intranasal administration (Alarcón-Arís et al., 2020, section 1.7.1).

#### Leucine-Rich Repeat Kinase 2

Altered LRRK2 activity has been associated with genetic and sporadic forms of PD (Jeong and Lee, 2020). As many of the pathogenic PD mutations in LRRK2 are gain of function, knockdown of LRRK2 may be desirable. Korecka et al. have shown that treating transgenic mice expressing human wild-type or G2019S LRRK2 with a single ICV injection of ASO induces LRRK2 exon 41 skipping and results in a decrease in phosphorylation of the LRRK2 kinase substrate RAB10. Exon 41 skipping also reverses LRRK2 kinase-dependent changes in LC3B II/I ratios, a marker for the autophagic process (Korecka et al., 2020).

#### Other NBT Targets in Parkinson’s Disease

Parkin functions as an E3 ligase in the ubiquitin–proteasome system and as a transcriptional repressor of p53. Pathogenic genomic deletions of PRKN exon 3 disrupt the reading frame and result in the loss of functional parkin protein, while deletions of both exon 3 and 4 maintain the reading frame and are associated with a later onset and milder disease. ASOs that induce exon 4 skipping to correct the reading frame restore functional parkin expression in PD patient cells with a heterozygous PRKN exon 3 deletion. The truncated semi-functional parkin isoform can be recruited to depolarized mitochondria and represses p53 transcription (L. Li et al, 2020).

Several of the proposed NBTs or gene therapy treatments can also be used in sporadic cases of PD as symptomatic treatments. The levodopa treatment in PD can be augmented by the enhanced expression of AADC that converts levodopa to dopamine. This route is now pursued by Voyager/Neurocrine. They are developing ezaladcigene resoparvovec (VY-AADC), an AAV2 vector expressing AADC under a CMV promoter, which is now in late clinical trials (Table 2). VY-AADC is administered through MRI-guided convective infusion using a posterior trajectory into the putamen, a brain region critical for neurotransmitter production. Long-term three-year data published in September 2020 demonstrated that a one-time treatment with VY-AADC led to significant improvement in motor function and quality of life in patients with PD. The improvement was sustained in 14 of 15 patients treated with VY-AADC three years ago (VY-AADC, 2020).

AXO-Lenti-PD is a lentiviral vector with an optimized expression cassette for the three dopamine biosynthesis enzymes (tyrosine hydroxylase, cyclohydrolase 1, and aromatic L-amino acid decarboxylase). It is delivered through 3 infusions/hemisphere to the postcommissural putamen using stereotactic guidance and is currently in clinical trials sponsored by Axovant for the treatment of PD in 4 patients and shows good tolerability and preliminary efficacy (Table 2; AXO-Lenti-PD, 2020).

Another therapeutic strategy in PD is the treatment with neurotrophic factors, for example, GDNF, known to enhance neuronal survival. AAV2-GDNF is now in clinical trial conducted by Brain Neurotherapy Bio, a joint effort between UCSF and Ohio State University (Table 2). AAV2-GDNF is administered through small openings on each side of the skull using stereotactic guidance to move a delivery cannula into the putamen, followed by a convection-enhanced delivery (CED) infusion assisted by intraoperative magnetic resonance imaging (iMRI) guidance and monitoring. Real-time iMRI during surgery allows for the distribution of the AAV2-GDNF to be tailored to the patient for optimal administration to precise brain regions. The study was supported by the California Institute for Regenerative Medicine created by the people of California to accelerate stem cell treatments to patients with unmet medical needs (AAV2-GDNF, 2020).

An earlier clinical study (NCT00985517, Table 2) for CERE-120, an AAV vector carrying neurturin, believed to be functionally similar to GDNF, conducted by Ceregene/Sangamo, has failed to show efficacy. CERE-120 was injected by a neurosurgeon directly into the substantia nigra and putamen. Interestingly, postmortem studies in two patients with advanced PD 8 and 10 years after CERE120 injection demonstrated persistent neurturin expression in the –3–12% cells of the putamen with dense tyrosine hydroxylase-positive fibers. In the substantia nigra, neurturin expression was detected in 9.8–39% of remaining melanin-containing neurons. There was no difference in the degree of Lewy pathology or in disease symptoms compared to untreated patients with PD. Similar results were obtained for other autopsy cases in these trials (at 1.5 and 3 months and for two subjects at 4 years post-administration). The authors propose that the lack of therapeutic effect may be due to insufficient dose of CERE-120 (Chu et al., 2020).

### Aromatic l-amino Acid Decarboxylase Deficiency

AADC (aromatic l-amino acid decarboxylase) deficiency (AADD) is caused by homozygous or compound heterozygous mutations in the DDC gene that result in combined serotonin and catecholamine deficiency, dystonia, and severe neurologic dysfunction. The estimated U.S. prevalence is approximately 1–3:100,000. Agilis/PTC is developing PTC-AADC, a functional DDC gene in an AAV vector that is injected intracerebrally into the putamen, a brain region critical for dopamine and serotonin production (Table 2). In three open-label clinical trials that enrolled a total of 26 children, treatment with PTC-AADC lowered the number of involuntary upward eye movements and led to the recovery of body weight and improvements in ability to sit, walk, and talk over a five-year period (PTC-AADC, 2020).

The prevalence of AADC deficiency in the Taiwanese population is higher than that of the world average due to the founder mutation IVS6+4A>T. In 2012–2017, 14 patients were treated with AAV-hAADC-2, initially developed for PD by Genzyme, with positive results. An open-label phase 1/2 study of AAV-hAADC-2 vector delivered *via* bilateral intraputaminal infusions in six patients was initiated in 2015–2017 in Taiwan. At up to 2 years after injection, the motor function, dystonia, and oculogyric crises were markedly improved in all patients. Treatment was more effective in younger patients (Kojima et al., 2019).

### Alzheimer’s Disease

Alzheimer’s disease (AD) is a neurodegenerative disorder that affects learning and memory. Although the prevalence of AD is about 5 million people in the United States, only approximately 10% of AD cases can now be traced to defined mutations in several genes, which brings the prevalence of each genetic type into orphan disease range. Heterozygous mutations in amyloid precursor protein (AAP), presenilin1 (PSEN1), and presenilin 2 (PSEN2) now account for the majority of familial cases with known etiology. Mutations in protein tau (MAPT), PLD3, TREM2, UNC5C, AKAP9, SORT1, and ADAM10 have also been associated with increased risk of AD (Kumar et al., 2020). Many of these genes were proposed as therapeutic targets for the treatment of both genetic and sporadic cases of AD.

Phosphorylated MAPT aggregates are believed to contribute to disease progression in AD and frontotemporal degeneration. Knockdown of MAPT has been shown to reduce MAPT aggregation, decrease neuronal death, and increase survival in preclinical models. IONIS-MAPTRx, a PS 2MOE ASO targeting MAPT in CNS, administered IT at 4-week intervals, is currently in a phase one clinical study in mild AD patients conducted by Ionis/Biogen (Table 2; IONIS-MAPTRx, 2020). Another MAPT-targeted NBT approach in AD is investigated by Sangamo/Biogen, using ST-501, an engineered zinc finger protein transcription factor, to repress MAPT expression. IND for clinical trials for ST-501 in Alzheimer’s is anticipated in 2021 (Sangamo, 2020).

Isoform e4 of apolipoprotein E (APOE) gene increases the risk of developing AD symptoms in both familial and sporadic forms of AD. Gene therapy approach for APOE-associated cases is being used in a clinical trial conducted by Weill Medical College of Cornell University and Alzheimer's Drug Discovery Foundation (NCT03634007). An AAVrh.10hAPOE2 vector-encoding human APOE2 allele is administered intracisternally in 15 participants with homozygous APOE4 alleles and AD (Table 2; Rosenberg et al., 2018).

Other therapeutic targets for NBTs in AD are now in the early preclinical stages of investigation. Low expression of one of the targets, brain-derived neurotrophic factor (BDNF), has been associated with AD and other disorders of the nervous system. Interfering with function of a BDNF NAT, a regulatory lncRNA in the BDNF locus, using ICV administration of ASOs (termed AntagoNATs), results in increased expression of biologically active BDNF protein in mouse brain and proliferation and differentiation of neurons in mouse neurosphere cultures (Modarresi et al., 2012, *Natural Antisense Transcripts*). Treatment with BDNF AntagoNAT targeted against BDNF-AS delivered using MIND technique induced widely distributed upregulation of BDNF protein in rat brain (Padmakumar et al., 2021).

Network dysrhythmias in AD and multiple neuropsychiatric disorders are associated with hypofunction of SCN1A, a voltage-gated sodium channel subunit predominantly expressed in interneurons. Increasing Nav1.1 levels in human amyloid precursor protein (hAPP)-expressing transgenic mice accelerated action potential kinetics of interneurons (Martinez-Losa et al., 2018). SCN1A levels can be upregulated using ASOs against SCN1A NAT (AntagoNATs). IT injections of AntagoNATs targeted against SCN1A NAT induced an increase in SCN1A levels in the brain of mice and nonhuman primates (Hsiao et al., 2016; *Dravet Syndrome*).

E3 ubiquitin ligase IDOL is a regulator of ApoE and β-amyloid metabolism. Genetic knockout of IDOL increases low-density lipoprotein receptor levels, which facilitates Aβ uptake and clearance by microglia. Treatment of APP/PS1 male mice with a *p*S/PD ASO with 5-methylcytosine, 2MOE, and cEt modifications, designed to reduce IDOL expression, reduced amyloid plaque load and neuritic dystrophy and improved the cognitive performance in Morris water maze (Gao et al., 2020).

CD33, a transmembrane sialic acid–binding receptor expressed on the surface of microglial cells, inhibits uptake and clearance of Abeta. ICV injection of an AAV vector encoding an artificial microRNA targeted against CD33 (miRCD33) in APP/PS1 mice reduced expression of pro-inflammatory genes (Tlr4, Il1b, Ccl2, and Tnfα) and amyloid beta accumulation (Griciuc et al., 2020).

TNFα may play a crucial role in the pathogenesis of AD and other neurodegenerative disorders due to its dual role as a pro-inflammatory mediator and as a negative regulator of fetal and adult neurogenesis and a positive regulator of neuronal differentiation (Hampel et al., 2020). In basal forebrain cholinergic neurons preferentially affected in AD, these effects may be mediated through changes in the expression of DNA-methylation enzymes (Guarnieri et al., 2020). Furthermore, TNF expression is known to be regulated by miRNA and lncRNA (D. Li et al, 2020; Jiang et al., 2020; Zhang et al., 2021). All of these mechanisms are easily accessible through NBTs.

### Neuronal Ceroid Lipofuscinoses

Neuronal ceroid lipofuscinoses (NCL or CLN) are a group of neurodegenerative disorders, sometimes collectively referred to as Batten disease that are characterized by the intracellular accumulation of autofluorescent lipopigment storage material. The disorders are currently classified numerically (CLN1-CLN13) according to the underlying gene alterations. Combined prevalence of all types of CLN is estimated at 1–9: 100 000. Gene therapy or NBT treatments for several of the Batten disease types are now in clinical trials (Johnson et al., 2019) and are briefly reviewed below.

#### Palmitoyl-Protein Thioesterase 1 (CLN1)

Abeona/Taysha is developing ABO-202, an scAAV9 vector carrying palmitoyl-protein thioesterase 1 (PPT1) gene for CLN1, which is caused by mutations in PPT1 (Table 2). The phase one/2 clinical trial of ABO-202 using a combined IV and IT delivery has received FDA clearance of its IND and is expected to start in 2021 (ABO-202, 2020).

#### Tripeptidyl Peptidase I (CLN2)

CLN2 is caused by recessive mutations in tripeptidyl peptidase I (TPP1). Although the FDA approved enzyme replacement drug cerliponase alfa (Brineura) developed by BioMarin for CLN2 in 2017, this treatment has to be administered repeatedly through ICV infusion. AAV-based NBTs can help reduce the administration route–related adverse effects.

CLN2 was the target of one of the early gene therapy clinical trials using AV2(CU)hCLN2 in 10 patients delivered by infusion into 12 distinct cerebral locations conducted by Weill Cornell and Nathan’s Battle Foundation (Souweidane et al., 2010). The study showed that viral delivery of the gene was safe and resulted in small but significant benefits to the patients. A more efficient vector system to deliver TPP1 is now being tested by Weill Cornell and NIH in a phase 1/2 trial with AAVrh.10CUhCLN2 (NCT01161576, NCT01414985; Table 2). The vector is delivered at 12 locations through six burr holes (2 locations at 2 depths through each hole), 3 burr holes per hemisphere.

REGENXBIO (Rockville, MD) expects to submit an IND for the intracisternal delivery of RGX-181, an AAV9 vector to deliver TPP1 gene for the treatment of CLN2, by the end of 2020, and plans to initiate enrollment in a phase I/II trial in the first half of 2021. Development of RGX-381, an AAV9 vector to deliver TPP1 gene directly to the retina for ocular manifestations of CLN2 disease, is also underway (RGX-181, 2020).

#### Battenin (CLN3)

CLN3, also known as battenin, is a membrane protein thought to be a component of the endosomal–lysosomal system. CLN3 deficiency has a prevalence of up to 1:25,000. Amicus is conducting a dose-escalation clinical trial to evaluate safety and efficacy of CLN3 gene therapy of AT-GTX-502 (scAAV9. P546. CLN3) delivered IT to the lumbar spinal cord for the treatment of CLN3 (Table 2; AT-GTX-502, 2020). Abeona is developing ABO-201, an AAV-based gene therapy for CLN3 disease that is currently in the preclinical stage (Abeona, 2020).

The most common mutation in CLN3 is a deletion of battenin exons 7 and 8 resulting in frameshift. An exon 5-targeted ASO was able to induce exon skipping and restore the open reading frame after a single injection in neonatal mice with this deletion. The exon skipping effect was observed for more than a year and was accompanied by improved motor coordination, reduced histopathology and increased survival (Centa et al., 2020).

#### Kufs Disease (CLN6)

CLN6 and CLN8 proteins, deficient in CLN6 (also known as Kufs disease) and CLN8 disorders, respectively, form a complex (termed EGRESS: ER-to-Golgi relaying of enzymes of the lysosomal system), which recruits lysosomal enzymes in the ER to facilitate their transfer to Golgi *via* COPII vesicles (Bajaj et al., 2020). Deficiency of CLN6 or CLN8 results in diminished levels of enzymes in the lysosomes.

Amicus Therapeutics has initiated clinical trials with AT-GTX-501, a CLN6 gene encoded by a self-complementary AAV9 (scAAV9), for Batten disease (CLN6 type). AT-GTX-501 is injected IT into the lumbar spinal cord region of subjects with mild-to-moderate CLN6 (Table 2).

#### MFSD8 Gene (CLN7)

MFSD8 is coding for a transporter protein involved in lysosomal movement and exocytosis. A pioneering case of a practical application of NBTs in personalized medicine is milasen (Table 1), a splice-modulating ASO designed for a single patient with Batten disease (CLN7 type) diagnosed based on lysosomal inclusions in skin biopsy. Genetic panel testing and whole-genome sequencing determined that the disease was caused by compound heterozygous mutations in MFSD8 gene: c.1102G→C and a previously unreported, approximately 2-kb aSINE–VNTR–Alu (SVA) retrotransposon insertion. The SVA insertion induced missplicing of MFSD8 exon six into a cryptic splice-acceptor site in MFSD8 intron six that caused premature translation termination. A 22-mer PS-2MOE ASO, designed to target the cryptic splice-acceptor site and nearby splice enhancers, boosted normal-to-mutant splicing ratios *in vitro* in patient cells by a factor of 2.5–3. After intrathecal injection in rats at therapeutic doses, milasen was well tolerated.

Clinical investigational treatment was initiated under an Expanded Access Investigational New Drug application. Eighteen grams of milasen drug substance was manufactured by TriLink Biotechnologies. Milasen was given by intrathecal bolus injection, starting at 3.5 mg and increasing approximately every 2 weeks up to 42 mg. After dose escalation, two additional loading doses were administered, followed by maintenance dosing approximately every 3 months. It took a year and 3 months from disease diagnosis to the administration of the 1st dose in the patient. Following treatment, frequency and duration of seizures decreased by greater than 50% than pretreatment levels. Neurologic and neuropsychological scores stabilized (Kim J et al., 2019).

#### CLN8 Gene (CLN8)

An scAAV9 vector carrying human CLN8 protein injected ICV in neonatal CLN8-deficient mice was well tolerated and produced robust CLN8 expression throughout the CNS from 4 to 24 months. At the same time, histopathological and behavioral hallmarks of the CLN disease were reduced, and the life span was increased from 10 months in untreated CLN8-deficient mice to beyond 24 months in treated animals (Johnson et al., 2020).

Multiple preclinical studies are now conducted to investigate possible therapies in other types of Batten disease.

### Mucopolysaccharidosis

Mucopolysaccharidosis (MPS) is a heterogeneous group of inherited disorders caused by abnormalities in genes involved in the metabolism of mucopolysaccharides. Several MPS subtypes are recognized, although these divisions do not always correspond to the causative gene. The prevalence of all forms of MPS is estimated to be 1:25,000. Although enzyme replacement and hematopoietic stem cell transplantation treatments are available in several MPS types, the enzymes do not cross the BBB and do not address the CNS aspects of the disease, while cell transplantation is an invasive method. As a result, development of NBTs could have a significant added benefit for patients with MPS.

#### Mucopolysaccharidosis Type I

Mucopolysaccharidosis type I (MPS I) includes Hurler syndrome, Hurler–Scheie syndrome, and Scheie syndromes that are caused by homozygous or compound heterozygous mutations in alpha-l-iduronidase (IDUA) gene that result in developmental abnormalities and intellectual disability.

REGENXBIO is testing RGX-111, an AAV9-based vector expressing IDUA, for the treatment of MPS I (Table 2). An investigator-initiated trial with RGX-111 conducted in a single patient dosed at the age of 21 months at CHOC Children’s has demonstrated increased IDUA activity, decreased heparan sulfate concentration, continued progression of neurocognitive development, and no drug-related adverse effects at 32 weeks postinjection. Recruitment has been initiated for phase I/II open label clinical trial evaluating RGX-111 delivered intracisternally for the treatment of MPS I (RGX-111, 2020). Sangamo is developing SB-318, a ZFN-targeted gene transfer of IDUA for the treatment of MPS I to be delivered IV, currently in phase 2 trial (Table 2).

#### Mucopolysaccharidosis Type II

Mucopolysaccharidosis type II (MPS II, Hunter syndrome) is caused by mutation in the gene encoding iduronate 2-sulfatase (IDS). The currently available enzyme replacement treatment idursulfase (Elaprase), a purified recombinant form of IDS administered IV, does not cross BBB and is ineffective against the CNS symptoms. Consequently, several gene therapy treatments for MPS II are now being developed.

REGENXBIO is conducting a phase 2 clinical trial of RGX-121, an AAV9 vector carrying a healthy copy of IDS administered intracisternally, in patients 5–18 years of age with MPS II (Table 2). RGX-121 was well tolerated in all six patients who received the treatment by fall of 2020. In parallel, the company has also initiated a prospective natural history study to collect data about the neurocognitive development of pediatric patients with MPS II, which they intend to share with the community (RGX-121, 2020). Sangamo is developing SB-913, a ZFN-targeted gene transfer of IDS for the treatment of MPS2 to be delivered IV, currently in the phase 2 trial (Table 2).

Avrobio is developing AVR-RD-05, cell therapy generated by modifying patient’s own hematopoietic stem cells using a lentiviral vector expressing IDS under control of proprietary tags, designed to optimize vector copy number, transduction efficiency, and resulting enzyme/protein activity. The cells modified with AVR-RD-05 can engraft in the bone marrow and generate daughter cells carrying the transgene that will potentially then engraft in the brain. An investigator-sponsored phase 1/2 clinical trial of AVR-RD-05 is anticipated to start in 2021 (AVR-RD-05, 2020).

#### Mucopolysaccharidosis IIIA

Mucopolysaccharidosis IIIA (Sanfilippo A syndrome) is caused by mutations in the N-sulfoglucosamine sulfohydrolase (SGSH) gene that degrades heparan sulfate. Accumulation of heparan sulfate in lysosomes leads to severe neurodegeneration and early death. One of the NBTs for Sanfilippo A syndrome is LYS-SAF302 that delivers a functional copy of the SGSH gene directly to brain cells using AAVrh.10, which has tropism for neurons. The treatment is administered through a one-time intracerebral infusion at six sites and is currently in clinical trials conducted by Lysogene (Table 2, section 1.10). In 2020, the FDA has issued a clinical hold on LYS-SAF302 trial, and in October, one of the patients in the study died. Currently, there is no evidence that the event is linked to the study drug administration (LYS-SAF302, 2020).

Another gene therapy drug delivering healthy SGSH gene using an AAV vector is ABO-102. It is administered through a one-time IV infusion and is now in phase I/II clinical trials in 15–22 patients conducted by Abeona (Table 2). Interim results from the trial show preservation of neurocognitive development and reduction in CSF heparan sulfate, a marker of increased SGSH activity in the CNS two years after treatment (ABO-101. ABO-102, 2020).

#### Mucopolysaccharidosis IIIB

Mucopolysaccharidosis IIIB (Sanfilippo Syndrome B) is caused by homozygous or compound heterozygous mutation in the gene encoding N-alpha-acetylglucosaminidase (NAGLU) and is characterized by severe CNS degeneration, but only mild somatic disease. UniQure Biopharma, Venn Life Sciences, and Institut Pasteur conducted clinical trials of rAAV2/5-hNAGLU delivered by a one-time intracerebral infusion into 16 sites (8 per hemisphere) with 4 participants (Tardieu et al., 2017; Table 2).

Abeona is conducting clinical trials of ABO-101 (rAAV9. CMV.hNAGLU) injected intravenously through a peripheral limb vein with 15 participants (Table 2). Interim results show that ABO-101 treatment improved multiple disease biomarkers in the 8 patients treated by the time of the analysis (ABO-101. ABO-102, 2020).

### Dravet Syndrome

Dravet syndrome (DS) is a severe childhood epilepsy accompanied by progressive psychomotor retardation and high incidence of sudden unexpected death. DS is caused by heterozygous loss-of-function mutations in the SCN1A gene, which encodes the pore-forming alpha subunit of the voltage-gated sodium channel Nav1.1. Majority of DS mutations lead to insufficient levels of SCN1A protein in inhibitory neurons where it is preferentially expressed (XY. Du et al, 2020). The resulting excitation/inhibition imbalance leads to network hyperexcitation and epileptic seizures, as well as other manifestations of DS (Scheffer and Nabbout, 2019). Small-molecule and device-based treatments for DS, approved and under clinical development, were reviewed recently (Samanta, 2020). As SCN1A mutations are not known to produce toxic protein, upregulation of the remaining healthy SCN1A allele represents a desirable therapeutic target in DS.

Another splicing-based approach utilizes the discovery of “toxic” exons naturally present in mRNAs produced by a large set of genes. Pre-mRNA molecules containing these exons cannot support translation and are destroyed through nonsense-mediated decay (Lim et al., 2020). Stoke Therapeutics is using splice-switching NBTs that can prevent the inclusion of toxic exons and as a consequence increase transcription of productive mRNAs. This approach is termed Targeted Augmentation of Nuclear Gene Output platform (TANGO) (Han et al., 2020). Stoke Therapeutics is currently conducting a phase 1/2a study of STK-001, an 2MOE PS ASO designed to skip a nonproductive exon in SCN1A gene, in Dravet syndrome (Table 2; Stoke, 2020).

Sarepta and StrideBio are in early discovery stages of gene therapy for DS (Sarepta, 2020).

SCN1A upregulation can be achieved by blocking the transcriptional inhibitory activity of a regulatory lncRNA from the SCN1A locus (SCN1ANAT) using 2OMe ASOs termed AntagoNATs (*Alzheimer’s Disease*). In nonhuman primates and a knock-in mouse model of Dravet, IT injection of AntagoNATs induced upregulation of Scn1a. A once-weekly injection of 20 μg of AntagoNAT for 4 weeks led to significant improvements in seizure frequency and duration, as well as normalization of excitability of hippocampal interneurons in Dravet mice (Hsiao et al., 2016). Studies with SCN1A AntagoNAT are now in the IND-enabling stage at OPKO Health.

Several therapeutics targeting biological pathways that could have an additive effect in increasing SCN1A are now in the early research stage. Overexpression of NaVβ1, an auxiliary subunit of the NAv1.1 channel using an AAV vector (AAV-NaVβ1), could facilitate the function of residual channels and improve the DS phenotype. A single AAV-NaVβ1 injection into the cerebral spinal fluid of neonatal male Scn1a ± mice led to increased survival, reduced spontaneous seizures, normalization of motor activity, and performance on the elevated plus maze test (Niibori et al., 2020).

Another potential adjunctive path for the treatment of DS is reduction of Scn8a transcript. In Scn1a ± mouse model of DS, treatment with an anti-Scn8a PS-2MOE ASO administered by ICV injection at postnatal day 2 resulted in Scn8a reduction of 25–50%. The treatment delayed seizure onset and extended survival of Dravet syndrome mice from 3 weeks to >5 months. Similar results were observed in a mouse with conditional Cre-dependent expression of a pathogenic mutation Scn8a-R1872W/+, a model of SCN8A encephalopathy. This gain-of-function mutation of SCN8A-containing sodium channel Nav 1.6 results in neuronal hyperactivity and seizures (Lenk et al., 2020).

Early research into gene editing in DS is currently used in mechanistic studies. Colasante et al. have identified single guide RNAs able to stimulate Scn1a transcription in association with the catalytically dead Cas9 activation system in cell lines, primary neurons, and mature DS interneurons. Scn1a protein levels were increased, and action potential firing was augmented. Scn1a-dCas9 activation system delivered to DS mouse pups using AAV attenuated febrile seizures and restored firing ability of parvalbumin GABAergic interneurons that are preferentially affected in DS (Colasante et al., 2020).

TALEN-mediated editing of the SCN1A gene was used to correct Q1923R mutation in SCN1A in Dravet patient iPSC cells (Zhao et al., 2020). Similar results were obtained using CRISPR/Cas9 gene editing. Two pairs of iPSCs were generated: one pair where a line from a GEFS + patient with the K1270T SCN1A mutation was corrected to control, and the other pair where a line from unaffected sibling was mutated to the K1270T mutation. In inhibitory and excitatory iPSC-derived neurons from these pairs, the K1270T mutation caused cell type–specific alterations in sodium current density and evoked firing, resulting in hyperactive neural networks (Xie et al., 2020).

Intravenous injections of AAV particles containing the optimal combination of 4 guide RNAs in the upstream, rather than downstream, promoter region into transgenic mice with Scn1a-haploinsufficiency and inhibitory neuron-specific expression of dCas9-VPR at four weeks of age increased Nav1.1 expression in parvalbumin-positive GABAergic neurons, ameliorated their febrile seizures, and improved their behavioral impairments (Yamagata et al., 2020).

### Angelman Syndrome

Angelman syndrome, characterized by developmental delay, seizures, and ataxia, is caused by maternal deficiency in the imprinted gene UBE3A (ubiquitin protein ligase E3A). The paternal copy of UBE3A is usually intact, but silenced by an lncRNA, UBE3A-ATS. The prevalence of AS is approximately 1:12,000 to 1:20,000. Several NBT approaches for AS are being investigated. ION582, a PS 2MOE ASO that targets UBE3A-ATS, is in preclinical stages of development for AS at Ionis (IONIS, 2020).

In 2020, Ovid Therapeutics and the University of Connecticut have announced a collaborative effort to expedite the development of miRNA vector (OV881) designed to block expression of UBE3A-ATS, an approach shown to induce derepression of the paternal copy of UBE3A (Meng et al., 2015).

As Angelman syndrome is associated with a reduction in tonic inhibition mediated by the delta-selective GABAA receptor; it can be used in combination with OV101 (gaboxadol), a small-molecule delta-selective GABAA receptor agonist that is currently being evaluated by Ovid in the pivotal phase three trial in Angelman syndrome (OV881, 2020).

Sarepta/Stride Bio is in early discovery stages of gene therapy for Angelman syndrome (Sarepta, 2020). Agilis/PTC Therapeutics: product pipeline also includes a gene therapy drug targeting Angelman syndrome (PTC, 2020).

### Alexander Disease

Alexander disease is a severe, progressive, and debilitating condition that eventually results in death due to loss of control over autonomic functions like breathing. The prevalence of Alexander disease is estimated at approximately 1:1,000,000. It is caused by mutations in glial fibrillary acidic protein (GFAP). Ionis is developing ION373, a PS 2MOE ASO designed to inhibit GFAP expression, now in the preclinical stage. In 2020, the European Medicines Agency (EMA) has granted orphan drug designation to ION373 for the treatment of Alexander disease (ION373, 2020).

### Canavan Disease

Canavan disease (CD) is caused by homozygous mutations in the ASPA gene that lead to accumulation of N-acetylaspartate (NAA) in the CNS and urine, spongiform degeneration of white matter, severe impairment of psychomotor development, and early death. The prevalence of CD in Ashkenazi Jewish population is approximately 1:10,000. Prevalence in other populations is unknown.

CD was the target of some of the earliest gene therapy studies (section 1.10). In a pilot study in two Canavan patients in 1996 using the-then state-of-the art gene therapy approach, a healthy copy of ASPA gene was injected through an intraventricular catheter attached to a plastic dome-shaped reservoir placed just beneath the scalp. After several improvements to expression vector and administration methods, to a large extent supported through fundraising efforts of Randell and Landsman families, the clinical trials for this therapy stalled due to lack of interest from the pharmaceutical companies which at the time were not pursuing rare disease indications and biologics and negative public perception of gene therapy studies (Canavan gene therapy, 2020). Only recently, further advances in viral vector design and understanding of the disease pathophysiology allowed to move the gene therapy for CD closer to the clinical stage (*Canavan Disease*).

This early work formed the basis for BBP-812, an AAV9 vector known to cross BBB developed by Aspa/BridgeBio in collaboration with UMASS Medical School, for the treatment of CD currently in the IND-enabling preclinical stage (BBP-812, 2020).

Recently, experiments in the AspA(−/−) mouse model of CD have shown that ICV administration of rAAVs carrying the Aspa gene prolonged survival compared to systemically delivered therapy, but, in contrast to systemic administration, failed to stabilize motor functions (Ahmed et al., 2016). Notably, most cells in the body are known to express ASPA and thus are likely to be affected by CD. Gessler et al. used an AAV9-based vector that can cross BBB when delivered systemically to introduce a codon-optimized ASPA with a Kozak sequence delivered IV and achieved early, complete, and sustained rescue of the lethal disease phenotype in CD mice. Interestingly, the treatment increased motor performance of both CD and WT mice beyond control WT levels (Gessler et al., 2017).

Alternative targets for NBTs in CD are also being investigated. As homozygous or heterozygous constitutive knockouts of Nat8l, an enzyme that synthesizes NAA and reduced disease severity in a CD disease model (AspaNur7/Nur7 mice), an NBT-mediated knockdown of this enzyme was attempted. Bannerman et al. (Bannerman et al., 2018) used AAV-Nat8l-shRNA, an AAV vector carrying a short-hairpin RNA against Nat8l sequence driven by the U6 promoter that was administered into the cerebral ventricles and cisterna magna of AspaNur7/Nur7 mice on postnatal day 1. The treatment suppressed disease phenotype. Hull et al. (Hull et al., 2020) used an LNA ASO designed to inhibit Nat8l administered into the cerebral ventricles of Aspa-deficient mice. The treatment reversed ataxia and diminished cerebellar and thalamic vacuolation and Purkinje cell dendritic atrophy.

### Friedreich’s Ataxia

Friedreich’s ataxia (FA) is a neurodegenerative disease characterized by progressive loss of movement and sensation. There are approximately 6,400 FA patients in the United States. FA is caused by a homozygous expanded trinucleotide AAG repeat in frataxin (FXN) gene intron that leads to reduced production of the frataxin protein. Voyager/Neurocrine is developing VY-FXN01, an AAVrh10 vector carrying a healthy FXN gene. The program is currently in preclinical development (Voyager, 2020).

Shen et al. have demonstrated that treatment with gapmer ASOs that are complementary to the expanded repeat can return the levels of FXN protein to near normal in patient-derived cell lines (Shen et al., 2020).

## Innovation in Clinical Trial Design and Drug Approval Procedures

As the number of patients affected by each of the orphan diseases is small and their condition is frequently severe, standard approaches to clinical trial design and statistical analysis of the results cannot be easily adapted to trials in orphan conditions. Furthermore, at the start of the trial, the natural history of these diseases is often not well studied, and reliable clinical trial readouts and biomarkers are not developed or tested, which also complicates the clinical trial design. As a result, an important component in the recent successes in developing NBTs for rare diseases was the progress in regulatory procedures and public participation.

Recently, the FDA has initiated expedited clinical development programs, including Fast Track, Priority Review, Accelerated Approval, Breakthrough Therapy, and Regenerative Medicine Advanced Therapy (RMAT). The EMA also has a program called the Innovation Task Force and an early access mechanism called PRIME (PRIority MEdicines), similar to the FDA’s Breakthrough Therapy.

The recently started Complex Innovative Trial Designs (CID) Pilot Program, an FDA initiative under the 21st Century Cures Act, is intended to promote innovation in this area that would be beneficial for the orphan disease trials (CID, 2020). The FDA will assist selected companies in using innovative trial design features, such as leveraging historical control data to augment the placebo arm, or use of Bayesian repeated measure modeling based on interim outcome analyses to develop a Bayesian disease progression models allowing adaptive trial design. Adaptive trial design incorporates historical control data and interim outcome data to predict the probability of clinical trial success and adjust enrollment numbers and study duration. A similar approach is utilized in sequential assignment trials, where the observations are assessed as they are produced and the total number of participants is not predetermined, but depends on the accumulated results. The subjects of the experimental group and the control group are enrolled in parallel such that the results for both groups can be examined as they accumulate. Furthermore, more attention is being paid to the development and use of composit endpoints for orphan diseases.

The Bridging Interventional Development Gaps (BrIDGs) program conducted by the NIH provides synthesis, formulation, pharmacokinetic and toxicology expertize, and resources. The NIH contractors conduct preclinical studies under the direction of the National Center for Advancing Translational Sciences (NCATS) staff. The decision to collaborate on a proposed project is based on an internal assessment of scientific merit, programmatic fit, and the availability of NIH funds. BrIDGs program has conducted harmacokinetic/absorption, distribution, metabolism, and excretion (PK/ADME), and IND-directed toxicology studies, clinical protocol development, and active ingredient manufacture for an NBT (EDN-OL1, a brain-penetrant NBT for Alzheimer’s disease) and several gene therapy drugs, including AAV2-AADC for AADC (aromatic l-amino acid decarboxylase) deficiency, sc-rAAV2.5IL-1Ra for osteoarthritis, and AAV2-GDNF for Parkinson’s disease (BrIDGs, 2020).

Nonprofit organizations, such as disease foundations that can support centralized patient registries, collect and conduct natural history trials, and Critical Path Institute (Critical Path Institute, 2020), a nonprofit organization focused on sharing data from the control arms of legacy clinical trials and developing consensus data standards, are important links in advancing the process of creating medicines for orphan diseases. The Critical Path Institute will also house the datasets from completed clinical trials. The modeling work and placebo data collected in the trials could be shared to accelerate progress in orphan disease treatment.

Although several the NBT clinical trials are already utilizing the adaptive/sequential trial designs (Table 2) and other innovations significantly more effort in this area is needed for continued success of NBTs in orphan diseases.

## Conclusion

The possibility of rational design and the resulting faster and more cost-efficient development cycles of NBTs have already fueled increased activity in the orphan disease field. However, significant problems still remain, and substantial further efforts are required to develop the scientific background and infrastructure for genetic testing, natural history studies and data sharing, NBT manufacturing, and regulatory support.

Better knowledge of the physiology and natural history of orphan diseases will be instrumental in biomarker identification and the choice of optimal methods for clinical monitoring for adverse events after NBT treatment.

Although severe adverse effects observed in several NBT trials may be related to the targeted biological mechanism, the possibility of toxicity associated with NBT chemistries, manufacturing procedures, or administration routes cannot be excluded. Accumulation of clinical experience with diverse targets and chemistries can help identify and eliminate chemistry-related toxicities.

The outcomes of the efforts in the orphan disease field will also benefit patients with “common” diseases through improved diagnostics, further development of the widely applicable NBT technology platforms, and innovative clinical trial protocols. Deeper understanding of biological mechanisms afforded by the orphan disease studies can elucidate common processes that underlie progression of related “common” diseases and expand therapeutic choices. Furthermore, with successes in genetic research, a growing proportion of “common” disease cases can now be assigned to mutations in particular genes, essentially extending the orphan disease field. Together, these developments in orphan diseases are building the foundation for future personalized medicine.
